# Prioritizing Environmental Chemicals for Obesity and Diabetes Outcomes Research: A Screening Approach Using ToxCast™ High-Throughput Data

**DOI:** 10.1289/ehp.1510456

**Published:** 2016-03-15

**Authors:** Scott Auerbach, Dayne Filer, David Reif, Vickie Walker, Alison C. Holloway, Jennifer Schlezinger, Supriya Srinivasan, Daniel Svoboda, Richard Judson, John R. Bucher, Kristina A. Thayer

**Affiliations:** 1 Division of the National Toxicology Program, National Institute of Environmental Health Sciences, National Institutes of Health, Department of Health and Human Services, Research Triangle Park, North Carolina, USA; 2 National Center for Computational Toxicology, Office of Research and Development, U.S. Environmental Protection Agency, Research Triangle Park, North Carolina, USA; 3 Bioinformatics Research Center, Department of Biological Sciences, North Carolina State University, Raleigh, North Carolina, USA; 4 Department of Obstetrics and Gynecology, McMaster University, Hamilton, Ontario, Canada; 5 Department of Environmental Health, Boston University School of Medicine, Boston, Massachusetts, USA; 6 Department of Chemical Physiology, The Scripps Research Institute, La Jolla, California, USA; 7 SciOme, LLC, Research Triangle Park, North Carolina, USA

## Abstract

**Background:**

Diabetes and obesity are major threats to public health in the United States and abroad. Understanding the role that chemicals in our environment play in the development of these conditions is an emerging issue in environmental health, although identifying and prioritizing chemicals for testing beyond those already implicated in the literature is challenging. This review is intended to help researchers generate hypotheses about chemicals that may contribute to diabetes and to obesity-related health outcomes by summarizing relevant findings from the U.S. Environmental Protection Agency (EPA) ToxCast™ high-throughput screening (HTS) program.

**Objectives:**

Our aim was to develop new hypotheses around environmental chemicals of potential interest for diabetes- or obesity-related outcomes using high-throughput screening data.

**Methods:**

We identified ToxCast™ assay targets relevant to several biological processes related to diabetes and obesity (insulin sensitivity in peripheral tissue, pancreatic islet and β cell function, adipocyte differentiation, and feeding behavior) and presented chemical screening data against those assay targets to identify chemicals of potential interest.

**Discussion:**

The results of this screening-level analysis suggest that the spectrum of environmental chemicals to consider in research related to diabetes and obesity is much broader than indicated by research papers and reviews published in the peer-reviewed literature. Testing hypotheses based on ToxCast™ data will also help assess the predictive utility of this HTS platform.

**Conclusions:**

More research is required to put these screening-level analyses into context, but the information presented in this review should facilitate the development of new hypotheses.

**Citation:**

Auerbach S, Filer D, Reif D, Walker V, Holloway AC, Schlezinger J, Srinivasan S, Svoboda D, Judson R, Bucher JR, Thayer KA. 2016. Prioritizing environmental chemicals for obesity and diabetes outcomes research: a screening approach using ToxCast™ high-throughput data. Environ Health Perspect 124:1141–1154; http://dx.doi.org/10.1289/ehp.1510456

## Introduction

The rise in obesity and diabetes rates are major threats to public health in the United States and abroad [Centers for Disease Control and Prevention (CDC) 2011; Dahlquist et al. 2011; DIAMOND Project Group 2006; Ogden and Carroll 2010; Patterson et al. 2009]. Excess caloric consumption and a sedentary lifestyle are well-recognized risk factors for obesity and diabetes. However, there is growing interest in the contribution of “nontraditional” risk factors to these conditions, including environmental chemicals. Research addressing the potential role of environmental chemicals in obesity and diabetes has rapidly expanded in the past several years, and the National Toxicology Program (NTP) has reviewed available information and identified research needs in this area (Behl et al. 2013; Maull et al. 2012; Taylor et al. 2013; Thayer et al. 2012).

One result of the reviews and studies conducted to date is the compilation of a list of molecular pathways whose disruption could increase the risk of obesity or diabetes. A logical step in the search for chemicals that could lead to these diseases is to examine *in vitro* data that indicate which chemicals may perturb the identified target pathways. To this end, we analyzed high-throughput screening (HTS) data from the U. S. Environmental Protection Agency (EPA) ToxCast™ program to identify candidate chemicals for consideration in future research on the environmental causes of obesity and diabetes. It is important not to equate perturbation of one of the diabetes-/obesity-associated pathways with a determination that a chemical causes obesity or diabetes. Bioactivity is one indicator that a chemical has the potential to alter a specific biological process, but whether that altered function produces a phenotypic outcome in an intact animal cannot be determined without further testing. Factors that can modulate the ultimate effects of bioactive chemicals include exposure, pharmacokinetics, diet, and the ability of an intact animal to compensate for the effects of perturbations at the molecular level.

In brief, our strategy was to *a*) solicit input from experts in the mechanisms of diabetes and obesity who participated in a 2011 NTP workshop, “Role of Environmental Chemicals in the Development of Diabetes and Obesity” (Thayer et al. 2012) to identify assay targets relevant to biological processes related to diabetes and obesity (e.g., insulin sensitivity in peripheral tissue, pancreatic islet and β cell function, adipocyte differentiation, and feeding behavior); and *b*) identify chemicals that perturb these targets or pathways. These chemicals then become candidates for future research. In this review, we describe the process of identifying pathways, the mapping of pathways to assays, and the identification of chemicals showing significant activity when tested in relevant HTS assays. A major goal of disseminating this information is to encourage the targeted follow-up research that is needed to assess the utility of HTS data for this type of activity.

## Methods

**Figure 1:**
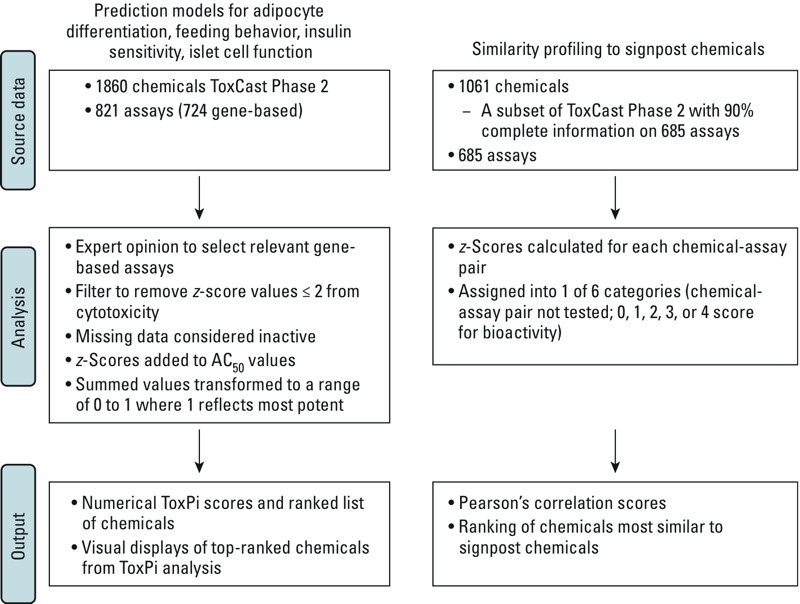
Analytical framework for source data and analyses.

An analytical framework to describe the methods described below is presented in Figure 1.

### Source *In Vitro* Data

In this review we analyzed data for an 1,860-compound ToxCast™ chemical library. The types of chemicals tested include pesticide actives and inert ingredients, industrial and consumer products, potential “green” chemicals that could be safer alternatives to existing chemicals, in-use and failed pharmaceuticals, and chemicals evaluated in NTP toxicity tests.

ToxCast™ currently provides results from ≤821 assay endpoints that make use of numerous technology platforms from 7 vendors (Kavlock et al. 2012) (see Excel File Table S1). These platforms include both cell-free (biochemical) and cell-based measures in multiple human primary cells, human or rodent cell lines, and rat primary hepatocytes (Judson et al. 2010). A wide spectrum of biological targets and effects is covered, including cytotoxicity, cell growth, genotoxicity, enzymatic activity, receptor binding, reporter genes, ion channels, transcription factor activity and downstream consequences, and high-content imaging of cells (Judson et al. 2010). Assays were performed by the individual vendors on uniformly procured chemical samples supplied by the U.S. EPA, and data were provided to the U.S. EPA for normalization and additional processing. In brief, chemicals were tested at 4–15 concentrations depending upon assay complexity, capacity, and cost. The data processing workflow by the U.S. EPA included normalization, curve fitting using Hill equations, visual examination of plots of the concentration–response relationships, and, finally, calculation of the concentration causing half-maximal response (AC50) or, in some platforms, the Lowest Effect Concentration (LEC). The specific criteria for determining the activity of a compound are platform-dependent and are described elsewhere (Kavlock et al. 2012). All analyses utilized the ToxCast™ data released in December 2014. In-depth information on the assays, the chemicals, and on ToxCast™ data processing can be accessed through the U.S. EPA website (http://actor.epa.gov/dashboard/).

### Expert Opinion–Based Approach to Identifying Relevant HTS Gene-Based Assays For Biological Processes

Many of the assays in ToxCast™ can be considered “gene-based” because the biochemical activity they assess is linked to a gene or to a set of genes (e.g., peroxisome proliferator-activated receptors α, δ, and γ; see Excel File Table S1 for a ToxCast™ assay list based on annotated gene names). Other assays are related to apical cellular phenotypes (e.g., cell death, mitochondrial damage) and are therefore too complex to map to a single gene or set of genes. In the current analysis, we sought to identify the gene-based assays relevant to the following biological processes related to diabetes or obesity: *a*) adipocyte differentiation, *b*) feeding behavior in rodents, *c*) feeding behavior in *Caenorhabditis elegans*, *d*) insulin sensitivity in peripheral tissue, *e*) pancreatic islet cell function, and *f*) pancreatic β cell function. With the exception of feeding behavior in rodents, the selected biological processes were considered to be appealing because of the availability of relatively inexpensive and rapid model systems (cell lines, *ex vivo*, short-term *in vivo*) that could be used to test hypotheses generated from the HTS results.

We consulted with topic-specific experts to identify relevant ToxCast™ gene-based assays for these biological processes (A.H., J.S., S.S., B. Blumberg, D. Clegg, and M. White). In brief, a list of the gene-based assays included in Phase I of ToxCast™ with annotated gene names (see Excel File Table S1) was distributed to several participants at the 2011 NTP workshop, “Role of Environmental Chemicals in the Development of Diabetes and Obesity” (Thayer et al. 2012). These experts individually selected the assays that they considered to be the most relevant to the biological processes listed above. The list of gene target assays chosen for each biological process is summarized in Table 1 and is listed by ToxCast™ assay names in Excel File Table S2.

**Table 1:** ToxCast™ assays included in each of the biological models.

ToxPi model inputs [ToxCast™ assays per input]		References
Adipocyte differentiation: 5 slices
PPARγ PPARγ: peroxisome proliferator-activated receptor gamma [4] PPRE: peroxisome proliferator-activated receptor response element [1]	RXRA RXRA: retinoid X receptor, alpha [1]	(Farmer 2006; Frijters et al. 2008; Hummasti et al. 2004; Janesick and Blumberg 2011; Mukherjee et al. 1997; Tontonoz et al. 1994; Wang 2010)
GR (or NR3C1): GR (or NR3C1): nuclear receptor subfamily 3, group C, member 1 (or glucocorticoid receptor) [4]	Other CEBPB: CCAAT/enhancer binding protein (C/EBP), beta [1] SREBF1: sterol regulatory element binding transcription factor 1 [1]	
LXR LXR: NR1H2 (or LXRB) - nuclear receptor subfamily 1, group H, member 2 (or liver X receptor) and NR1H3 (or LXRA) - nuclear receptor subfamily 1, group H, member 3 [2] LXRE: LXR response element [1]		
Feeding behavior (rodents): 9 slices
CCK: cholecystokinin A and B receptors [2]	INSR: insulin receptor [2]	(Barros and Gustafsson 2011; Deblois and Giguère 2011; Frijters et al. 2008; Ranhotra 2010; Skibicka and Dickson 2013)
ESR1: estrogen receptor α or 1 [4]	MAP: mitogen-activated protein kinase 3 [3]	
ESRRA: estrogen-related receptor alpha [1]	NPY: NPY neuropeptide Y receptors Y1, Y2, Y5; Bos taurus [3]	
FoxO1: forkhead box O1 [1]	STAT3: signal transducer and activator of transcription 3 (acute-phase response factor) [1]	
IL6: interleukin 6 (interferon, beta 2) [1]		
Feeding behavior (*C. elegans*): 12 slices
DRD2: dopamine receptor D2 [1]	INSR: insulin receptor [2]	(de Bono and Bargmann 1998; Frijters et al. 2008; Noble et al. 2013; Sawin et al. 2000; Srinivasan 2015; Srinivasan et al. 2008)
GSK3B: glycogen synthase kinase 3 beta [1]	NPY: NPY neuropeptide Y receptors Y1, Y2, Y5; Bos taurus [3]	
HTR2C: 5-hydroxytryptamine (serotonin) receptor 2C [1]	PPARδ: peroxisome proliferator-activated receptor delta [1]	
HTR3A: 5-hydroxytryptamine (serotonin) receptor 3A [1]	PRKACA: protein kinase, cAMP-dependent, catalytic, alpha [1]	
HTR2A: 5-hydroxytryptamine (serotonin) receptor 2A [1]	Sstr1: somatostatin receptor 1 [1]	
Other HTR Slc6a4: solute carrier family 6 (neurotransmitter transporter, serotonin), member 4 [2] Htr1a: 5-hydroxytryptamine (serotonin) receptor 1A [Mus musculus] [1] Htr4: 5-hydroxytryptamine (serotonin) receptor 4 [1] HTR6: 5-hydroxytryptamine (serotonin) receptor 6 [1] HTR7: 5-hydroxytryptamine (serotonin) receptor 7 (adenylate cyclase-coupled) [1] HTR5A: 5-hydroxytryptamine (serotonin) receptor 5A [1]	Other PPARγ: peroxisome proliferator-activated receptor gamma [4] PPRE: peroxisome proliferator-activated receptor response element [1] DRD4: dopamine receptor D4 [1] DRD1: dopamine receptor D1 [1] NR1I1: vitamin D (1,25-dihydroxyvitamin D3) receptor [2] NR1H2 (or LXRB): nuclear receptor subfamily 1, group H, member 2 (or liver X receptor) and NR1H3 (or LXRA) - nuclear receptor subfamily 1, group H, member 3 [2] NR1H3 (or LXRA): nuclear receptor subfamily 1, group H, member 3 (Liver X receptor alpha) [1] CEBPB: CCAAT/enhancer binding protein (C/EBP), beta [1]	
Insulin sensitivity in peripheral tissue: 11 slices
AKT: v-akt murine thymoma viral oncogene homolog 1 [2]	PPARγ: peroxisome proliferator-activated receptor γ [4]	(Frijters et al. 2008; Wang 2010)
CREB: cAMP responsive element binding protein 3 [1]	PPRE: peroxisome proliferator-activated receptor response element [1]	
FOX: forkhead box A2; forkhead box O1 [2]	PTPN1: protein tyrosine phosphatase, nonreceptor type 1 [1]	
INSR: insulin receptor [2]	SREBF1: sterol regulatory element binding transcription factor 1 [1]	
Kcnj11: potassium inwardly rectifying channel, subfamily J, member 1 [1]	STAT3: signal transducer and activator of transcription 3 (acute-phase response factor) [1]	
PPARα: peroxisome proliferator-activated receptor alpha [2]		
Islet cell function: 11 slices
betaCatenin: TCF/b-catenin response element [1]	INSR: insulin receptor [2]	(Frijters et al. 2008; Greeley et al. 2011)
DRD1: DRD dopamine receptors D1, D2, D3, D5 [Bos taurus] [1]	Kcnj11: potassium inwardly-rectifying channel, subfamily J, member 1 [1]	
FOXA2: forkhead box A2 [1]	ONECUT1: one cut homeobox 1 [1]	
FOXO1: forkhead box O1 [1]	PAX6: paired box 6 [1]	
GSK3B: glycogen synthase kinase 3 beta [1]	PTPN1: protein tyrosine phosphatase, nonreceptor type 1 [1]	
HNF4A: hepatocyte nuclear factor 4, alpha [1]		
β cell function: 14 slices
ACHE: acetylcholinesterase [2]	HRT, solute carrier: solute carrier family 6 (neurotransmitter transporter, serotonin), member 4 [2]	(Amireault et al. 2013; Barros and Gustafsson 2011; Caicedo 2013; Eldor et al. 2013; Frijters et al. 2008; Greeley et al. 2011; Gupta et al. 2010; Tiano and Mauvais-Jarvis 2012; Ustione et al. 2013; Wang 2010)
BCHE: butyrylcholinesterase [1]	INSR: insulin receptor [2]	
DRD: dopamine receptors (multiple subtypes) and opioid receptor, delta 1 [5]	Kcnj11: potassium inwardly-rectifying channel, subfamily J, member 1 [1]	
DRD, solute carrier: [ 2]	PPARα: peroxisome proliferator-activated receptor alpha [2]	
ESR1: estrogen receptor α or 1 [4]	PPARδ: peroxisome proliferator-activated receptor delta [1]	
GABA: gamma-aminobutyric acid (GABA) receptor (multiple subtypes) [5]	PPARγ: peroxisome proliferator-activated receptor γ [4]	
HTR: 5-hydroxytryptamine (serotonin) (multiple subtypes) [8]	PPRE: peroxisome proliferator-activated receptor response element [1]	
Frijters et al. (2008) was used in a 2010 analysis conducted for the NTP workshop “Role of Environmental Chemicals in the Development of Diabetes and Obesity” (Thayer et al. 2012), where CoPub text-mining tools were used to identify relationships between genes, pathways/processes, diseases, and drugs. The relationship is summarized in CoPub with an R-scale score that quantifies the strength of cocitation between two keywords (e.g., PNPLA3 and fatty liver). In the 2010 analysis, CoPub was searched for genes associated with adipocyte differentiation, feeding behavior, insulin sensitivity, and islet cell function, and the results were mapped to the ToxCast™ assay targets listed above. The CoPub analysis was considered to provide support for an association between the gene and the biological process when the R-scale score was ≥ 25. Many, but not all, of the gene targets identified by expert opinion were identified in the CoPub analysis.		

### ToxPi Analysis of Biological Process Models

We calculated a ToxPi score for each biological process–chemical pair using data from ToxCast™.

The ToxPi framework (Filer et al. 2014; Reif et al. 2013) was used to create these scores based on the ToxCast™ data for each of the six biological processes selected by the experts. The combination of the genes, assays, and scores for a biological process is called the “biological process model.” Each component of the score for a biological process model (a slice in the ToxPi visualization) was equally weighted so that each component/slice had the same potential contribution to the score. It is important to emphasize that this approach only identifies chemicals with predicted absolute effects on these biological pathways and does not necessarily identify the direction of the effect in terms of potentially adverse or therapeutic: for example, pharmaceuticals used to treat diabetes would be expected to affect relevant biological pathways.

The input values for the ToxPi analysis were calculated as follows from the AC_50_ (concentration at half-maximal activity) and the *z*-score (the distance from cytotoxicity; higher *z*-scores indicate increased potency from the chemical-specific cytotoxicity distribution) values provided in the December 2014 ToxCast™ release. First, the AC_50_ values were transformed to negative log molar units. For example, an active chemical–assay pair with an AC_50_ value of 1 μM would have a negative log–transformed value of 6. Second, inactive chemical–assay pairs or chemical–assay pairs with a *z*-score ≤ 2 were assigned values of 0. Third, for active chemical–assay pairs, the *z*-score was added to the transformed AC_50_ value. For example, a chemical–assay pair with an AC_50_ value of 1 μM and a *z*-score of 5.4 would have an input value of 11.4 (transformed AC_50_ value of 6 + *z*-score value of 5.4).

Exclusion of chemical–assay pairs with *z*-score values ≤ 2 accounts for a phenomenon referred to as the “cytotoxic signal burst,” which manifests itself as an increase in nonspecific assay activation near concentrations where cell stress and cytotoxicity occur (R. Judson, personal communication). Selecting a cutoff value of 2 eliminates the majority of what appear to be cell stress/cytotoxicity-related false positive activities in the assay data while retaining marginal or ambiguous hits (R. Judson, personal communication). To positively weight more specific responses (higher potency relative to cytotoxicity), the *z*-scores were added to their respective potency values.

Briefly, the ToxPi scores were calculated by summing the input values across all assays in a component/slice for each chemical. The summed values of the individual assays were then transformed to range from 0 to 1 by subtracting the minimum value and dividing by the range. The values were then multiplied by the proportional weight for that component/slice (1 divided by the number of slices for equally weighted slices, as presented here) to give the component score. The final ToxPi score was calculated by summing each component score, and ranged from 0 to 1, where a ToxPi score of 1 would mean that chemical was the most potent chemical in each component/slice of the model. Note that because some regions of the data matrix are sparse, this approach is only useful for an initial identification of candidate positive chemicals but will miss others for which testing data are not available.

### “Signpost” Chemicals for Metabolic Disorders Included in Phase 2 of ToxCast™

To provide context for the HTS data, we compared the screening results for several chemicals included in ToxCast™ to findings from the published literature. To identify signpost chemicals, we used a previous summary provided as background material for the 2011 NTP workshop “Role of Environmental Chemicals in the Development of Diabetes and Obesity” (National Toxicology Program, see “Literature Review Documents,” Thayer et al. 2012) or as documented in clinical observations of drug effects (Dang et al. 2005; Sheehan 2005). The following chemicals tested in ToxCast™ were used as signpost chemicals: troglitazone, tributyltin chemicals, nicotine, haloperidol and chlorpromazine, tolazamide, amitraz, dexamethasone, nicotinic acid (niacin), and chlorinated persistent organic pollutants (POPs). Other environmental chemicals of interest, such as bisphenol A and phthalates, were not considered signpost chemicals because of uncertainties related to the consistency and/or interpretation of findings at the time of the 2011 NTP workshop or in a subsequent systematic review (Kuo et al. 2013; Maull et al. 2012; Taylor et al. 2013). To the best of our knowledge, high-quality reviews (i.e., reviews that adhere to systematic review methodology and reporting standards) have not been published more recently than those mentioned above. However, bisphenol A, phthalates, and other environmental chemicals studied for metabolic effects that were included in ToxCast™ (including metabolites and other members of the same chemical class) are highlighted in the ToxPi graphics.

Chemical concordance could not be evaluated using a more systematic comparison because diabetes and obesity-related outcomes are not standard end points in toxicological studies; therefore, these end points are not available for the majority of the environmental chemicals or drugs tested in ToxCast™. In addition, a number of environmental chemicals and drugs associated with diabetes, weight gain, or other metabolic effects have not yet been tested in ToxCast™, including atypical antipsychotics (Taylor and McAskill 2000), arsenic (Maull et al. 2012), and certain organochlorine chemicals (Taylor et al. 2013).

### Chemical–Chemical Correlation Analysis

To complement the scores for the specific biological processes, a correlation analysis was performed for each chemical–chemical pair across all ToxCast™ assays within the subset of ToxCast™ chemicals that had the most complete testing coverage (1,061 of the 1,860 chemicals across 685 of the 821 assays). Unlike the biological process analysis, the correlation analysis was limited to the subset of the ToxCast™ chemicals with the most complete testing coverage (ToxCast™ Phase I and II chemicals) to minimize the impact of missing data in the correlation profiles. Pearson’s correlation values for each chemical–chemical pair were calculated on complete pair-wise observations using only transformed *z*-score values (see below) from each assay. This approach compares the assay-specific profiles of the chemicals across all assays. In addition, we note that this analysis is independent of the genes and pathways that were annotated to assays and used in the above-mentioned metabolic disease biological process models. The *z*-score values were transformed by binning values into six categories, with the last four indicating increasing specificity of the metabolic bioactivity:

Chemical-assay pairs not tested = N/A
Tested, inactive or only tested at single concentration and presumed inactive = 0
Tested, active, and *z*-score ≤ 3 = 1
Tested, active, and 3 < *z*-score ≤ 6 = 2
Tested, active, and 6 < *z*-score ≤ 9 = 3
Tested, active, and *z*-score > 9 = 4

This procedure provided, for each chemical, a list of chemicals ranked by overall bioassay similarity across the larger ToxCast™ assay suite as a way to complement the biological process models. The similarity profiling provided a list of additional candidate chemicals to consider for targeted research and could potentially provide the basis for developing chemotypes for metabolic disorders.

### Chemical Clustering Based Upon ToxPi Similarity

Principal components analysis (PCA) was performed on the feeding behavior (*C. elegans*) ToxPi output matrix to illustrate an approach for identifying similar clusters of compounds. First, we selected all principal components (PCs) that explained ≥ 5% of the overall variance. Second, we performed *k*-means clustering on the reduced PCs matrix using 10,000 iterations and a maximum number of clusters (*k*) equal to the dimensions of the reduced PCs matrix. Third, we plotted the PCs of each chemical as points colored by cluster, plus the mean ToxPi profiles of each cluster.

### Calculations

All calculations and analyses were performed using R (R Core Team 2014). Source data are available at http://www.epa.gov/chemical-research/toxicity-forecaster-toxcasttm-data, and R-code is available as supplemental material (R-scripts folder).

## Results

### Overview of Relative Biological Process Model Results

The top 30 chemicals for each biological process model are listed in Table 2 (also shown as ToxPi graphics in Figures S1–S6). The biological process model scores for all 1,860 chemicals are available in Excel File Tables S3–S8, where chemicals can be sorted by overall score for a given biological process model or for individual components/slices: for example, peroxisome proliferator-activated receptor gamma (PPARγ) or glucocorticoid receptor (GR) activity. These tables also contain information on chemical properties (i.e., logP, estimated percent human oral absorption), which can be used to further prioritize targeted follow-up research efforts. In Figures S1–S6, we also indicate how other chemicals of high research interest for metabolic effects, such as bisphenol A, phthalate metabolites, perfluorooctane sulfonate (PFOS), perfluorooctanoic acid (PFOA), and several organophosphates and their metabolites, ranked in our analysis. In many cases, heavily studied chemicals (or metabolites) were not included in the top 30 chemicals for the biological processes. The chemical structures represented in the top 30 lists for each biological process are diverse (see Excel File Table S18, Figures S9–S12).

**Table 2:** Top 30 chemicals [CASRN] based on prediction model scores for adipocyte differentiation, feeding behavior (rodent), feeding behavior (*Caenorhabditis elegans*), insulin sensitivity in peripheral tissue, islet cell function, and beta cell function.

Rank	Adipocyte differentiation	Feeding behavior (rodents)	Feeding behavior (*C. elegans*)	Insulin sensitivity peripheral tissue	Islet cell function	Beta cell function
1	Diallyl phthalate [131-17-9] (score = 0.306, *RAR score = 0.115) Use: plasticizer Class: phthalate	HMR1171 [328392-46-7] (score = 0.192) Use: pharmaceutical Class: lipid lowering	Chlorpromazine hydrochloride [69-09-0] (score = 0.258) Use: pharmaceutical Class: dopamine antagonist	Farglitazar [196808-45-4] (score = 0.250) Use: pharmaceutical Class: PPARγ agonist	Isopropyl triethanolamine titanate [36673-16-2] (score = 0.177) Use: coupling Class: organometallic	Raloxifene hydrochloride [82640-04-8] (score = 0.240) Use: pharmaceutical Class: selective estrogen receptor modulator (SERM)
2	Methyl salicylate [119-36-8] (score = 0.293, *RAR score = 0.180) Use: flavor, antiseptic Class: salicylate	PharmaGSID_48511 [1062243-51-9] (score = 0.133) Use: pharmaceutical Class: polo-like kinase inhibitor	Trelanserin [189003-92-7] (score = 0.241) Use: pharmaceutical Class: selective serotonin 5-HT2A, Antagonist	PharmaGSID_47315 [444610-91-7] (score = 0.221) Use: pharmaceutical Class: PPARγ agonist	Basic blue 7 [2390-60-5] (score = 0.176) Use: dye Class: aniline dye	PharmaGSID_47315 [444610-91-7] (score = 0.225) Use: pharmaceutical Class: PPARγ agonist
3	Melengestrol acetate [2919-66-6] (score = 0.289) Use: pharmaceutical Class: steroidal progestin	4-Hydroxytamoxifen [68392-35-8] (score = 0.125) Use: pharmaceutical Class: SERM	Fabesetron hydrochloride [129299-90-7] (score = 0.239) Use: pharmaceutical Class: serotonin 5-HT3 receptor antagonist	Basic blue 7 [2390-60-5] (score = 0.204) Use: dye Class: aniline dye	PharmaGSID_48511 [1062243-51-9] (score = 0.130) Use: pharmaceutical Class: polo-like kinase inhibitor	SSR150106 [NOCAS_47362] (score = 0.223) Use: pharmaceutical Class: chemokine receptor antagonist
4	Rotenone [83-79-4] (score = 0.266, *RAR score = 0.222) Use: insecticide Class: botanical	Niclosamide [50-65-7] (score = 0.114) Use: molluscicide Class: phenol halide	Volinanserin [139290-65-6] (score = 0.236) Use: pharmaceutical Class: serotonin 5-HT2A receptor antagonist	Rotenone [83-79-4] (score = 0.203) Use: insecticide Class: botanical	Spiromesifen [283594-90-1] (score = 0.106) Use: insecticide Class: phenyl tetronic acid	PharmaGSID_47259 [149062-75-9] (score = 0.221) Use: pharmaceutical Class: acetylcholinesterase inhibitor
5	Tebufenpyrad [119168-77-3] (score = 0.257, *RAR score = 0.120) Use: insecticide Class: pyrazole	PharmaGSID_47337 [1061517-62-1] (score = 0.112) Use: pharmaceutical Class: cholecystokinin 1 receptor (CCK1R) agonist	Isopropyl triethanolamine titanate [36673-16-2] (score = 0.225) Use: coupling Class: organometallic	Tebufenpyrad [119168-77-3] (score = 0.197) Use: insecticide Class: pyrazole	Tris(2,3-dibromopropyl) phosphate [126-72-7] (score = 0.100) Use: flame retardant Class: phosphate alkyl halide	Farglitazar [196808-45-4] (score = 0.203) Use: pharmaceutical Class: PPARγ agonist
6	*Trans*-retinoic acid [302-79-4] (score = 0.251, *RAR score = 1) Use: pharmaceutical Class: carboxylic acid	Acetic acid C8-10-branched alkyl esters, C9-rich [108419-33-6] (score = 0.111) Use: solvent Class: carboxylate	SSR150106 [NOCAS_47362] (score = 0.222) Use: pharmaceutical Class: chemokine receptor antagonist	PharmaGSID_48511 [1062243-51-9] (score = 0.187) Use: pharmaceutical Class: polo-like kinase inhibitor	Apigenin [520-36-5] (score = 0.097) Use: flavone Class: genistein-like	Chlorpromazine hydrochloride [69-09-0] (score = 0.192) Use: pharmaceutical Class: dopamine antagonist
7	Isazofos [42509-80-8] (score = 0.248) Use: insecticide Class: organophosphate	Methyl parathion [298-00-0] (score = 0.111) Use: insecticide Class: organophosphate	PharmaGSID_48511 [1062243-51-9] (score = 0.174) Use: pharmaceutical Class: polo-like kinase inhibitor	Isopropyl triethanolamine titanate [36673-16-2] (score = 0.182) Use: coupling Class: organometallic	Resorcinol [108-46-3] (score = 0.091) Use: intermediate, disinfectant Class: phenol	UK-416244 [402910-27-4] (score = 0.189) Use: pharmaceutical Class: selective serotonin reuptake inbitor (SSRI)
8	Aspirin [50-78-2] (score = 0.246, *RAR score = 0.071) Use: pharmaceutical Class: phenyl carboxylic acid alkoxy	Isopropyl triethanolamine titanate [36673-16-2] (score = 0.111) Use: coupling Class: organometallic	SB243213A [200940-23-4] (score = 0.148) Use: pharmaceutical Class: serotonin 5-HT2C receptor inverse agonist	Pyridaben [96489-71-3] (score = 0.177) Use: insecticide Class: diazine phenyl sulfide halide ketone	Acetic acid C8-10-branched alkyl esters, C9-rich [108419-33-6] (score = 0.091) Use: solvent Class: carboxylate	Volinanserin [139290-65-6] (score = 0.176) Use: pharmaceutical Class: serotonin 5-HT2A receptor antagonist
9	GW473178E methyl benzene sulfonic acid [263553-33-9] (score = 0.221) Use: pharmaceutical Class: thrombin inhibitor	Ilepatril [473289-62-2] (score = 0.111) Use: pharmaceutical Class: vasopeptidase inhibitor	Haloperidol [52-86-8] (score = 0.142) Use: pharmaceutical Class: dopamine inverse agonist	1,3-Diphenyl-1,3-propanedione [120-46-7] (score = 0.165) Use: plasticizer Class: phenyl	Haloperidol [52-86-8] (score = 0.091) Use: pharmaceutical Class: dopamine inverse agonist	Haloperidol [52-86-8] (score = 0.163) Use: pharmaceutical Class: dopamine inverse agonist
10	Bentazone [25057-89-0] (score = 0.221) Use: herbicide Class: carbamate	Equilin [474-86-2] (score = 0.111) Use: pharmaceutical Class: steroidal estrogen	PharmaGSID_47315 [444610-91-7] (score = 0.132) Use: pharmaceutical Class: PPARγ agonist	Fenamiphos [22224-92-6] (score = 0.156) Use: insecticide Class: organophosphate	Dibenz[*a*,*h*]anthracene [53-70-3] (score = 0.091) Use: research Class: polycyclic aromatic hydrocarbon	PharmaGSID_48511 [1062243-51-9] (score = 0.149) Use: pharmaceutical Class: polo-like kinase inhibitor
11	Sodium abietate [14351-66-7] (score = 0.215) Use: coating Class: abietate	Triisononyl trimellitate [53894-23-8] (score = 0.111) Use: plasticizer Class: phthalate	Elzasonan [361343-19-3] (score = 0.130) Use: pharmaceutical Class: selective 5-HT1B and 5-HT1D receptor antagonist	2,4,6-Trichlorophenol [88-06-2] (score = 0.153) Use: herbicide, fungicide, reactant Class: chlorinated phenol	Caffeine [58-08-2] (score = polycyclic aromatic hydrocarbon) Use: pharmaceutical; natural Class: Not Assigned caffeine-like	*meso*-Hexestrol [84-16-2] (score = 0.145) Use: pharmaceutical Class: nonsteroidal estrogen
12	2-Ethyl-2-hexenal [645-62-5] (score = 0.212) Use: intermediate, insecticide Class: aldehyde	Cymoxanil [57966-95-7] (score = 0.110) Use: fungicide Class: acetamide carboxylate amine	Raloxifene hydrochloride [82640-04-8] (score = 0.126) Use: pharmaceutical Class: SERM	(*Z*,*E*)-Fenpyroximate [111812-58-9] (score = 0.151) Use: insecticide Class: pyrazole	*N*-nitrosodipropylamine [621-64-7] (score = 0.091) Use: breakdown product, research Class: nitrosoamine	Trelanserin [189003-92-7] (score = 0.135) Use: pharmaceutical Class: serotonin 5-HT2A antagonist
13	AVE8923 [NOCAS_47381] (score = 0.207) Use: pharmaceutical Class: tryptase inhibitor	AVE6324 [NOCAS_47377] (score = 0.108) Use: pharmaceutical Class: factor Xa inhibitor	Allura red C.I.16035 [25956-17-6] (score = 0.117) Use: dye Class: phenyl sulfuric acid dye	2-Ethyl-2-hexenal [645-62-5] (score = 0.150) Use: intermediate, insecticide Class: aldehyde	Dicyclopentadiene [77-73-6] (score = 0.091) Use: intermediate Class: alkene	2,2-Bis(4-hydroxyphenyl)-1,1,1-trichloroethane [2971-36-0] (score = 0.128) Use: degradate Class: phenol halide
14	SR271425 [155990-20-8] (score = 0.205, *RAR score = 0.084) Use: pharmaceutical Class: thioxanthone analog	Zearalenone [17924-92-4] (score = 0.108) Use: mycotoxin Class: carboxylic acid ketone	AVE6324 [NOCAS_47377] (score = 0.108) Use: pharmaceutical Class: factor Xa inhibitor	Diuron [330-54-1] (score = 0.146) Use: herbicide Class: phenyl urea	Rotenone [83-79-4] (score = 0.091) Use: insecticide Class: botanical	4-Hydroxytamoxifen [68392-35-8] (score = 0.123) Use: pharmaceutical Class: SERM
15	Tributyltin benzoate [4342-36-3] (score = 0.200, *RAR score = 0.145) Use: microbicide Class: organotin	Rifampicin [13292-46-1] (score = 0.102) Use: pharmaceutical Class: antibiotic	SSR241586 [NOCAS_47353] (score = 0.103) Use: pharmaceutical Class: 2,2-disubstituted morpholine	Propargite [2312-35-8] (score = 0.137) Use: insecticide Class: phenyl ether sulfate yne	2,4,6-Trichlorophenol [88-06-2] (score = 0.091) Use: herbicide, fungicide, reactant Class: phenol halide	17α-Ethinylestradiol [57-63-6] (score = 0.122) Use: pharmaceutical Class: steroidal estrogen
16	Farglitazar [196808-45-4] (score = 0.200) Use: pharmaceutical Class: PPARγ agonist	Mestranol [72-33-3] (score = 0.101 Use: pharmaceutical Class: nonsteroidal estrogen	Mercuric chloride [7487-94-7] (score = 0.103) Use: bactericide Class: organometallic	1-(6-*tert*-Butyl-1,1-dimethyl-2,3-dihydro-1H-inden-4-yl)ethanone [13171-00-1] (score = 0.130) Use: fragrance Class: phenyl ketone	Silica [7631-86-9] (score = 0.087) Use: filler Class: silicate	9-Octadecenoic acid, 12-hydroxy-, (9*Z*,12*R*) [141-22-0] (score = 0.122) Use: pharmaceutical, natural, plasticizer Class: unsaturated omega-9 fatty acid
17	Acrylamide [79-06-1] (score = 0.193) Use: reactant Class: acrylamide	*meso*-Hexestrol [84-16-2] (score = 0.098) Use: pharmaceutical Class: steroidal estrogen	Calcium neodecanoate [27253-33-4] (score = 0.099) Use: additive Class: carboxylic acid	Isoxaben [82558-50-7] (score = 0.129) Use: herbicide Class: amide, oxazole	Tannic acid [1401-55-4] (score = 0.086) Use: natural Class: phenol benzoic acid	Calcium neodecanoate [27253-33-4] (score = 0.121) Use: additive Class: carboxylic acid
18	1-(6-*tert*-Butyl-1,1-dimethyl-2,3-dihydro-1H-inden-4-yl)ethanone [13171-00-1] (score = 0.180, *RAR score = 0.142) Use: fragrance Class: phenyl ketone	Estriol [50-27-1] (score = 0.097) Use: pharmaceutical Class: steroidal estrogen	FD&C Yellow 6 [2783-94-0] (score = 0.09) Use: dye Class: phenyl sulfuric acid dye	1,4-Diaminoanthraquinone [128-95-0] (score = 0.129) Use: dye Class: anthraquinone	Perfluorooctane sulfonate, PFOS [1763-23-1] (score = 0.086) Use: fluorosurfactant Class: perfluoro sulfuric acid	2,4,6-Trichlorophenol [88-06-2] (score = 0.120) Use: herbicide, fungicide, reactant Class: chlorinated phenol
19	Tetrabutyltin [1461-25-2] (score = 0.177, *RAR score = 0.157) Use: microbicide Class: organotin	Pirimiphos-methyl [29232-93-7] (score = 0.097) Use: insecticide Class: organophosphate	Aspirin [50-78-2] (score = 0.093) Use: pharmaceutical Class: nonsteroidal antiinflammatory drugs (NSAIDs)	Sodium abietate [14351-66-7] (score = 0.127) Use: coating Class: abietate	1-Phenoxy-2-propanol [770-35-4] (score = 0.085) Use: pesticidal inert, solvent Class: phenol ethoxylate alcohol	17α-Estradiol [57-91-0] (score = 0.1120) Use: pharmaceutical Class: steroidal estrogen
20	Triamcinolone [124-94-7] (score = 0.172) Use: pharmaceutical Class: corticosteroid	Diethylstilbestrol [56-53-1] (score = 0.095) Use: pharmaceutical Class: nonsteroidal estrogen	Diphenhydramine hydrochloride [147-24-0] (score = 0.090) Use: pharmaceutical Class: antihistamine (“Benadryl”)	Glyceryl monoricinoleate [1323-38-2] (score = 0.127) Use: intermediate, emulsifier Class: alcohol carboxylate	Dimethyl succinate [106-65-0] (score = 0.084) Use: intermediate Class: carboxylate	Diphenhydramine hydrochloride [147-24-0] (score = 0.119) Use: pharmaceutical Class: antihistamine (“Benadryl”)
21	Pyridaben [96489-71-3] (score = 0.171, *RAR score = 0.063) Use: insecticide Class: diazine phenyl sulfide halide ketone	Raloxifene hydrochloride [82640-04-8] (score = 0.095) Use: pharmaceutical Class: SERM	PD 0343701 [676116-04-4] (score = 0.089) Use: pharmaceutical Class: dopamine D2 receptor, 5HT2A	Troglitazone [97322-87-7] (score = 0.125) Use: pharmaceutical Class: thiazolidinediones	1,3-Diphenyl-1,3-propanedione [120-46-7] (score = 0.083) Use: plasticizer Class: phenyl	Glyceryl monoricinoleate [1323-38-2] (score = 0.119) Use: intermediate, emulsifier Class: alcohol carboxylate
22	Resorcinol [108-46-3] (score = 0.167, *RAR score = 0.162) Use: intermediate, disinfectant Class: phenol	17beta-Estradiol [50-28-2] (score = 0.095) Use: pharmaceutical Class: steroidal estrogen	SSR240612 [NOCAS_47351] (score = 0.087) Use: pharmaceutical Class: kinin B1 receptor antagonist	Isazofos [42509-80-8] (score = 0.123) Use: insecticide Class: organophosphate	Auramine hydrochloride [2465-27-2] (score = 0.082) Use: dye, disinfectant Class: aniline	Clomiphene citrate [50-41-9] (score = 0.117) Use: pharmaceutical Class: SERM (“Clomid”)
23	Dexamethasone sodium phosphate [2392-39-4] (score = 0.160) Use: pharmaceutical Class: corticosteroid	4,4'-Methylenedianiline [101-77-9] (score = 0.093) Use: intermediate Class: aniline	Farglitazar [196808-45-4] (score = 0.084) Use: pharmaceutical Class: PPARγ agonist	Famoxadone [131807-57-3] (score = 0.123) Use: fungicide Class: dicarboximide	Dibenzothiophene [132-65-0] (score = 0.081) Use: fragrance, flavor Class: benzofuran	17β-Estradiol [50-28-2] (score = 0.115) Use: pharmaceutical Class: steroidal estrogen
24	Phenobarbital sodium [57-30-7] (score = 0.160) Use: pharmaceutical Class: barbituate	Pyraflufen-ethyl [129630-19-9] (score = 0.092) Use: herbicide Class: pyridine alkoxy carboxylic acid halide	Fomesafen [72178-02-0] (score = 0.084) Use: herbicide Class: diphenyl ether	HMR1171 [328392-46-7] (score = 0.122) Use: pharmaceutical Class: lipid lowering	2,4,6-Tribromophenol [118-79-6] (score = 0.080) Use: intermediate, antiseptic Class: phenol halide	SAR150640 [NOCAS_47389] (score = 0.114) Use: pharmaceutical Class: β3-adrenoceptor agonist
25	CP-457677 [214535-77-0] (score = 0.159) Use: pharmaceutical Class: not assigned	17α-Ethinylestradiol [57-63-6] (score = 0.092) Use: pharmaceutical Class: steroidal estrogen	Diallyl phthalate [131-17-9] (score = 0.084) Use: plasticizer Class: phthalate	Pirinixic acid [50892-23-4] (score = 0.121) Use: pharmaceutical Class: PPARα agonist	Sulfasalazine [599-79-1] (score = 0.080) Use: pharmaceutical Class: sulfa drug	*N*-dodecanoyl-*N*-methylglycine [97-78-9] (score = 0.111) Use: cosmetic, surfactant Class: carboxylic acid amide
26	Basic blue 7 [2390-60-5] (score = 0.156) Use: dye Class: aniline dye	Nicotine [54-11-5] (score = 0.090) Use: pharmaceutical, pesticide Class: pyridine amine	Methyl parathion [298-00-0] (score = 0.083) Use: insecticide Class: organophosphate	Dinocap [39300-45-3] (score = 0.120) Use: fungicide Class: dinitrophenol derivative	1-(6-*tert*-Butyl-1,1-dimethyl-2,3-dihydro-1H-inden-4-yl)ethanone [13171-00-1] (score = 0.078) Use: fragrance Class: phenyl ketone	Pyrimethamine [58-14-0] (score = 0.111) Use: pharmaceutical Class: protozoal infections, antimalarial drug
27	(*Z*,*E*)-Fenpyroximate [111812-58-9] (score = 0.156, *RAR score = 0.235) Use: insecticide Class: pyrazole	Benzal chloride [98-87-3] (score = 0.088) Use: dye, reactant Class: phenyl halide	*N*-nitrosodipropylamine [621-64-7] (score = 0.083) Use: breakdown product, research Class: nitrosoamine	*Z*-tetrachlorvinphos [22248-79-9] (score = 0.119) Use: insecticide Class: organophosphate	Chlorpromazine hydrochloride [69-09-0] (score = 0.078) Use: pharmaceutical Class: dopamine antagonist	2-Naphthalenol [135-19-3] (score = 0.111) Use: antioxidant, reactant Class: naphthalene alcohol
28	CP-612372 [353280-07-6] (score = 0.155) Use: pharmaceutical Class: not assigned	Methyleugenol [93-15-2] (score = 0.088) Use: fragrance, flavor, attractant, anesthetic Class: phenol ethoxylate alkyl	Trioctyl trimellitate [89-04-3] (score = 0.083) Use: plasticizer Class: phthalate	Apigenin [520-36-5] (score = 0.118 Use: flavone Class: genistein-like	PharmaGSID_48505 [NOCAS_48505] (score = 0.077) Use: pharmaceutical Class: CDK2 inhibitor	Propylparaben [94-13-3] (score = 0.104) Use: microbicide Class: paraben
29	2-Methyl-5-nitroaniline [99-55-8] (score = 0.155) Use: intermediate Class: aniline nitro	17α-Estradiol [57-91-0] (score = 0.085) Use: pharmaceutical Class: steroidal estrogen	Rifampicin [13292-46-1] (score = 0.083) Use: pharmaceutical Class: antibiotic	Sodium dodecyl sulfate [151-21-3] (score = 0.118) Use: surfactant Class: sulfuric acid alkyl	Ethyl butyrate [105-54-4] (score = 0.075) Use: flavor Class: carboxylate	SAR377142 [NOCAS_47385] (score = 0.103) Use: pharmaceutical Class: factor Xa inhibitor
30	Retinol acetate [127-47-9] (score = 0.154) Use: natural; vitamin Class: carboxylate	Piperazine [110-85-0] (score = 0.085) Use: insecticide Class: amine	Resorcinol [108-46-3] (score = 0.082) Use: intermediate, disinfectant Class: phenol	Sodium 2,4,7-tri(propan-2-yl)naphthalene-1-sulfonate [1323-19-9] (score = 0.118) Use: pesticide other, adjuvant Class: naphthalene sulfuric acid	Tolazamide [1156-19-0] (score = 0.074) Use: pharmaceutical Class: sulfonylurea	Pirinixic acid [50892-23-4] (score = 0.102) Use: pharmaceutical Class: PPARα agonist

Pharmaceuticals were among the highest-scoring chemicals, and their known mechanistic target(s) were often identified in ToxCast™. For example, the dopaminergic activities of haloperidol and chlorpromazine hydrochloride (both antipsychotic medications) and the PPARγ activity of farglitazar (a PPARγ agonist developed for treatment of hepatic fibrosis) were detected.

Of the top 200 ranked chemicals in the adipocyte differentiation process, 138 were identified as having retinoic acid receptor (RAR) agonist activity by the same methods as those described for other assays included in the adipocyte model. These chemicals may not stimulate adipocyte differentiation because activation of RAR can block downstream signaling (Bonet et al. 2012; Frey and Vogel 2011). Researchers interested in using the prioritization results from the adipocyte differentiation prediction process (see Figure S1) should also review the RAR ToxCast™ activity data presented in Excel File Table S3 (see column T, “RAR_score”).

### Chemical Clustering Based upon a Model of Feeding Behavior in *C. Elegans*

PCA followed by *k*-means clustering was used to illustrate how ToxPi output can be translated into multidimensional similarity scores of activity across slices. Figure S13 shows the mean ToxPi profiles of feeding behavior in *C. elegans* for the three clusters with the highest overall ToxPi scores (see Table 1 for an explanation of the component assays in each slice). The 24 chemicals with the highest average ToxPi scores (“Cluster 3”) were characterized by activity on slices representing Other, OtherHTR, HTr1, DRD2, and HTR2C, such as chlorpromazine hydrochloride. The 11 chemicals with the second-highest average ToxPi scores (“Cluster 2”) were characterized by activity on slices representing Other and NPY, such as the pharmaceutical AVE6324. The 15 chemicals with the third-highest average ToxPi scores (“Cluster 7”) were characterized by activity on slices representing Other, PPARd, and INSR, such as the pharmaceutical PharmaGSID_47315. The remaining clusters further partition the variation within ToxPi scores into clusters of similar activity, including a large cluster of 1,200 chemicals representing negligible (or no) activity in this model.

### Signpost Chemicals

Most of the signpost chemicals (10 of 12 chemicals or classes of chemicals) would have been prioritized as chemicals of interest using a criterion of being in the top 10% most highly ranked in one or more biological process models. Most organochlorine chemicals included in ToxCast™ would not have been prioritized because they were not ranked highly in any biological process (DDT isomers, heptachlor expoxide, mirex, dieldrin, lindane), and nicotinic acid and β-hexachlorocyclohexane ranked in the top 15% of only one biological process; therefore, they likely would not be flagged as chemicals of high interest. In the following sections, we discuss the findings for each signpost chemical (or chemical class) in detail.

***Signpost chemicals prioritized in the prediction models.* Troglitazone.** Troglitazone is an antidiabetic drug that decreases insulin resistance by increasing adipocyte differentiation via activation of PPARγ (Sheehan 2005). Its use has been associated with weight gain in humans, and it is used as a positive control compound in cellular models of adipogenesis (another widely used positive control compound, rosiglitazone, is not currently included in the ToxCast™ library). The PPARγ activity of troglizazone was identified in ToxCast™, and it was ranked highly (in the top 5–10%) for adipocyte differentiation, feeding behavior (*C. elegans*), insulin sensitivity, and in the biological process models for β cell function (Table 3). Chemicals that have similar activity to troglitazone across the ToxCast™ assay set are shown in Table 4, and the full correlation analysis set is available in Excel File Table S9.

**Table 3:** Rank of signpost chemicals out of 1,860 chemicals included in ToxCast™.

Compound [CASRN]	Adipocyte differentiation	Feeding behavior (rodent)	Feeding behavior (*Caenorhabditis elegans*)	Insulin sensitivity	Islet cell function	β cell function
Amitraz [33089-61-1] Use: insecticide; Class: formamidine	—	134**	113**	419	149**	320
Chlorpromazine hydrochloride [69-09-0] Use: pharmaceutical (conventional antipsychotic); Class: phenyl halide	666	—	1***	490	27***	6***
Haloperidol [52-86-8] Use: pharmaceutical (conventional antipsychotic); Class: phenyl-phenyl [COCnN] halide alcohol	423	502	9***	399	7***	9***
Nicotine [54-11-5] Use: pharmaceutical/ pesticide other/ natural; Class: pyridine amine	—	26***	—	—	—	—
Nicotinic acid (niacin) [59-67-6] Use: vitamin; Class: pyridine carboxylic acid	—	—	—	—	—	—
Dexamethasone sodium phosphate [CASRN2392-39-4] Use: pharmaceutical (synthetic corticosteroid); Class: steroid	23**	—	—	—	—	—
Tributyltin benzoate [4342-36-3] Use: microbicide; Class: organometallic	15***	455	135**	154**	197*	146**
Tributyltin chloride [1461-22-9] Use: microbicide; Class: organometallic	69***	81***	257	114**	218*	103**
Tributyltin methacrylate [2155-70-6] Use: microbicide; Class: organometallic	112**	430	449	322	199*	354
Tolazamide [1156-19-0] Use: pharmaceutical (antidiabetic drug); Class: phenyl sulfonamide amine	—	276*	—	112**	30***	93**
Troglitazone [97322-87-7] Use: pharmaceutical (antidiabetic drug); Class: not assigned	51***	—	108**	21***	—	69***
Persistant organochlorines^*a*^						
*p*,*p'*-DDE [72-55-9]	—	—	—	—	—	—
*p*,*p'*-DDT [50-29-3]	—	460	—	—	—	917
*o*,*p'*-DDT [789-02-6]	—	272*	689	—	—	699
Heptachlor epoxide [1024-57-3]	—	—	—	—	—	—
Mirex [2385-85-5]	—	—	—	—	—	—
Dieldrin [60-57-1]	—	—	—	—	—	—
β-Hexachlorocyclohexane (β-HCH) [319-85-7]	—	206*	—	—	—	614
Lindane (γ-HCH) [58-89-9]	—	—	707	635	—	481
^***a***^Dichlorodiphenyltrichloroethane (DDT) or dichlorodiphenyldichloroethylene (DDE). *** (pink squares) In top ~5th percentile. ** (blue squares) In top ~10th percentile. * (green squares) In top ~15th percentile. —Not active, score = 0.						

**Table 4:** Similarity analysis: Top 10 most similar nonpharmaceuticals in ToxCast™ (rank ordered by Pearson correlation of *z*-score values).

Rank	Amitraz	Haloperidol	Nicotine	Dexamethasone	Tributyltin chloride	Tolazamide	Troglitazone
1	Diquat dibromide monohydrate 0.337 [6385-62-2] Herbicide	Gentian violet 0.439 [548-62-9] Fungicide	Mepiquat chloride 0.553 [24307-26-4] Herbicide	Cyclohexanol 0.292 [108-93-0] Precursor	Tributyltin methacrylate 0.859 [2155-70-6] Microbicide	Sucrose 0.477 [57-50-1] Sweetener	Quinoxyfen 0.511 [124495-18-7] Herbicide
2	Tralkoxydim 0.307 [87820-88-0] Herbicide	Difenzoquat metilsulfate 0.405 [43222-48-6] Herbicide	Imidacloprid 0.430 [138261-41-3] Insecticide	1,3-Dichloro-5,5-dimethylhydantoin 0.268 [118-52-5] Disinfectant, reactant	Triphenyltin hydroxide 0.517 [76-87-9] Fungicide	Butylbenzene 0.477 [104-51-8] Plasticizer, solvent, surfactant	Dichlorprop 0.476 [120-36-5] Herbicide
3	Pentamidine isethionate 0.288 [140-64-7] Microbicide	1-Benzylquinolinium chloride 0.4018 [15619-48-4] Industrial	Triisononyl trimellitate 0.428 [53894-23-8] Plasticizer	Benzoic acid 0.265 [65-85-0] Intermediate, preservative	Gentian violet 0.500 [548-62-9] Fungicide	4-Aminofolic acid 0.395 [54-62-6] Rodenticide	Dihexyl phthalate 0.464 [84-75-3] Plasticizer
4	*N*-phenyl-1,4-benzenediamine 0.258 [101-54-2] Intermediate	Didecyldimethylammonium chloride 0.388 [7173-51-5] Bactericide	Acetamiprid 0.328 [135410-20-7] Insecticide	2-Phenoxyethanol 0.253 [122-99-6] Intermediate, fragrance, solvent	Phenylmercuric acetate 0.492 [62-38-4] Fungicide	4-Nitrotoluene 0.351 [99-99-0] Reactant	3,3',5,5'-Tetrabromo-bisphenol A 0.442 [79-94-7] Flame retardant
5	FD&C yellow 5 0.256 [1934-21-0] Dye	Pentamidine isethionate 0.387 [140-64-7] Microbicide	Thiacloprid 0.299 [111988-49-9] Insecticide	Pentaerythritol 0.249 [115-77-5] Explosives/weapons	Didecyldimethylammonium chloride 0.483 [7173-51-5] Bactericide	Methenamine 0.348 [100-97-0] Intermediate	Oxadiazon 0.434 [19666-30-9] Herbicide
6	Mercuric chloride 0.252 [7487-94-7] Bactericide	Mercuric chloride 0.382 [7487-94-7] Bactericide	Clothianidin 0.276 [210880-92-5] Insecticide	Clove leaf oil 0.237 [8000-34-8] Natural	Octhilinone 0.444 [26530-20-1] Fungicide	Pyrithiobac-sodium 0.343 [123343-16-8] Herbicide	Clotrimazole 0.412 [23593-75-1] Fungicide
7	Difenzoquat metilsulfate 0.248 [43222-48-6] Herbicide	Tributyltin methacrylate 0.373 [2155-70-6] Microbicide	Nitrobenzene 0.251 [98-95-3] Reactant	1-Tetradecanol 0.229 [112-72-1] Intermediate	Mercuric chloride 0.395 [7487-94-7] Bactericide	4-Vinyl-1-cyclohexene dioxide 0.335 [106-87-6] Pesticide, reactant	Spirodiclofen 0.408 [148477-71-8] Insecticide
8	FD&C yellow 6 0.246 [2783-94-0] Dye	Dodecyltrimethylammonium chloride 0.369 [112-00-5] Bactericide	Biphenyl 0.247 [92-52-4] Intermediate, fungicide	Sodium saccharin hydrate 0.228 [82385-42-0] Additive	1,2-Benzisothiazolin-3-one 0.383 [2634-33-5] Fungicide	Novaluron 0.321 [116714-46-6] Insecticide	Octrizole 0.408 [3147-75-9] UV absorber
9	1,2-Benzisothiazolin-3-one 0.238 [2634-33-5] Fungicide	*N*-methyldioctylamine 0.354 [4455-26-9] Reactant	2,6-Dimethylphenol 0.238 [576-26-1] Intermediate	4,4'-Bipyridine 0.216 [553-26-4] Degradate	2,4-Bis(1-methyl-1-phenylethyl)phenol 0.381 [2772-45-4] Intermediate	Etridiazole 0.316 [2593-15-9] Fungicide	Butralin 0.404 [33629-47-9] Herbicide
10	Forchlorfenuron 0.227 [68157-60-8] Plant growth regulator	Tributyltin chloride 0.334 [1461-22-9] Microbicide	2-Butoxyethanol 0.238 [111-76-2] Solvent	Diacetone alcohol 0.214 [123-42-2] Solvent	Ziram 0.377 [137-30-4] Fungicide	2,4,6-Trichlorophenol 0.297 [88-06-2] Herbicide, fungicide, reactant	2,4-Bis(1-methyl-1-phenylethyl)phenol 0.399 [2772-45-4] Intermediate

**Tributyltin chemicals.** Trisubstituted organotins, such as tributyltin (TBT), were previously used as biocides for antifouling paints to slow the growth of aquatic organisms, but they are now extremely restricted for use in inland waterways. TBT has been shown to stimulate adipocyte differentiation (*in vitro* and *in vivo*) and to increase the amount of fat tissue in adult animals exposed to TBT during fetal life or weaning (Grün and Blumberg 2006; Kirchner et al. 2010) and transgenerationally in the F3 generation following direct treatment to the F0 generation (Chamorro-García et al. 2013). TBT is a potent agonist for both PPARγ and retinoid X receptor alpha (RXRα), two receptors that heterodimerize and are known to promote adipocyte differentiation *in vitro* when activated (Grün et al. 2006). It should be noted that the *in vitro* profiles of the tin compounds are among the most complex of any of the compounds tested, with hundreds of assays being activated.

The biological process models identified tributyltin compounds, in the form of tributyltin benzoate, tributyltin methacrylate, and tributyltin chloride, as chemicals of interest (Table 3). ToxCast™ also detected interactions with dopaminergic, adrenergic, and serotonin receptors at relatively low concentrations (AC_50_ ≤ 10 μM) for tributyltin chloride and tributyltin methacrylate (data not shown). Chemicals exhibiting similar patterns of activity to those of tributylin chloride are shown in Table 4. The full correlation analysis sets for tributyltin chloride and tributyltin methacrylate are available in Excel File Tables S10 and S11 (tributyltin benzoate was not included in the chemical set used for correlation analyses).

The adipogenic effects of TBT associated with PPARγ and RXRα activation have been documented, but its effects on insulin sensitivity have not been throughly explored. It should be noted that a diabetic phenotype for triphenyltin (TPT) has been reported in the literature (see “Organotins and Phthalates Literature Review Documents,” Thayer et al. 2012). Studies suggest that rats and mice may be relatively insensitive models for studying the effects of organotins on glucose regulation (Zuo et al. 2011) and that rabbits and hamsters may be more sensitive (Matsui et al. 1984; Ohhira et al. 1999). The diabetic phenotype appears to be transient (Ogino et al. 1996), with no histological abnormalities noted in the islet cells (Matsui et al. 1984; Miura et al. 1997). Implicated mechanisms include reduction of [Ca(2+)](i) and insulin secretion in response to K(ATP) channel-dependent depolarization, and related decreases of NAD(P)H and ATP production during glucose metabolism in pancreatic islet cells (Miura et al. 1997, 2012; Miura and Matsui 2001, 2006; Watanabe et al. 2002).

**Nicotine.** Nicotine is a parasympathomimetic agent that is present in the nightshade family of plants. It acts as a pharmacological stimulant through the activation of nicotinic acetylcholine receptors. Inhaling tobacco smoke from either active or passive (e.g., second-hand smoke) smoking is the main source of nicotine exposure for the general population (CDC 2013). Epidemiological data support a positive association between maternal smoking and increased risk of obesity or overweight in children after infancy (Behl et al. 2013; Ino 2010; Oken et al. 2008). These data were considered strongly suggestive of a causal relationship by participants in the 2011 NTP workshop and are supported by findings from animal studies (Behl et al. 2013). The association with obesity or overweight following exposure during development is different from effects that occur with exposure later in life, where smoking is known to suppress appetite, and adult smokers tend to gain weight after smoking cessation (Yang et al. 2013; Zoli and Picciotto 2012). Rats exposed to nicotine during perinatal development tended to have higher body weight and more fat mass compared with controls; typically, the effect first became apparent at weaning and persisted through adulthood (Behl et al. 2013). The mechanism(s) by which nicotine might be acting are not well established, but studies have suggested that nicotine alters brain circuitry by affecting leptin signalling in the hypothalamus; a role is also implicated for central hypothyroidism induced by a hypothalamic–pituitary dysfunction (Behl et al. 2013; see “Maternal Smoking During Pregnancy/Nicotine Literature Review Documents,” Thayer et al. 2012). The feeding behavior in rodent models identified nicotine as a chemical of interest (ranked 26, in the top 5% of chemicals) (Table 3). The nicotine metabolite cotinine did not rank highly in any biological process model.

Nicotine was considered active on three assay targets at an AC_50_ of < 10 μM, binding to human nicotinic cholinergic receptor, alpha 2 (CHRNA2) and rodent cholinergic receptor, nicotinic, alpha 7 (Chrna7) at AC_50_ values of 0.62 and 1.69 μM, respectively, and up-regulating estrogen-related receptor alpha (ERRα, or gene symbol ESRRA) at an AC_50_ value of 3.39 μM. The high rank of nicotine for feeding behavior in rodents was based mostly on interactions with ERRα. Although the binding interactions with nicotinic cholinergic receptors were expected, the interaction with ERRα has not been previously identified and is of interest given the apparent role of ERRs in regulating adipogenesis, energy homeostasis, diabetes, and heart disease (Bonnelye and Aubin 2013; Deblois and Giguère 2011; Ju et al. 2012; Ranhotra 2010; Villena and Kralli 2008). Chemicals exhibiting the greatest similarity in activity across the ToxCast™ assays are shown in Table 4, and the full correlation analysis set is available in Excel File Table S12.

**Haloperidol and chlorpromazine.** Haloperidol and chlorpromazine are primarily used for the treatment of schizophrenia and have been associated with weight gain in patients (Musil et al. 2015; Sheehan 2005). The effects of these drugs on both schizophrenia and weight gain appear to be mediated through a blockade of a number of G-protein coupled receptors that mediate the effects of serotonin, histamine, and dopamine. Individuals taking haloperidol experience increased appetite and sedation along with a decrease in basal metabolic rate. Both haloperidol and chlorpromazine were ranked in the top 5% most active chemicals in biological process models for feeding behavior in *C. elegan*s and in models for islet and β cell function (Table 3), suggesting that these chemicals would have been effectively prioritized for potential effects on metabolic function. Chemicals with activity profiles similar to that of haloperidol are shown in Table 4. The full correlation analysis sets for both haloperidol and chlorpromazine are available in Excel File Tables S13 and S14, respectively.

**Tolazamide.** Tolazamide is a sulfonylurea drug used to treat diabetes. Sulfonylureas have been associated with hypoglycemia and weight gain in patients (Dang et al. 2005; Sheehan 2005). As pharmaceuticals, sulfonylurea derivatives help control diabetes by increasing insulin secretion from β cells, which results in a lowering of blood glucose. More specifically, sulfonylureas bind with high affinity to the sulfonylurea receptor-1 subunit (SUR1) of the ATP-sensitive potassium channel [K(ATP)] in pancreatic β cells (Thévenod 2002). Sulfonylurea binding causes K(ATP) channels to close, reducing potassium conductance and leading to membrane depolarization. Membrane depolarization leads to the opening of calcium channels and the entry of Ca^+2^ ions into the β cell, which then triggers insulin secretion and a subsequent decrease in blood glucose levels (NLM 2014). The stimulation of insulin secretion by sulfonylureas, though beneficial in the short term, may cause pancreatic damage because of overstimulation, which may in turn cause an increase in reactive oxygen species, endoplasmic reticulum stress, mitochondrial dysfunction, and β cell death (Remedi and Nichols 2008). Although SUR1 is not included in ToxCast™, tolazamide was ranked in the top 5–10% in the islet and β cell function models, in the top 10% for insulin sensitivity, and in the top 15% for feeding behavior in rodents (Table 3). The high ranking of tolazamide in the insulin sensitivity, islet cell, and β cell models was based on binding assay results for the ATP-sensitive potassium inwardly rectifying channel (KCNJ11) gene, which is commonly associated with diabetes of genetic origin (Greeley et al. 2011). K(ATP) channels are found in the cell membranes of pancreatic β cells and open and close in response to blood glucose levels. At least 30 mutations in the KCNJ11 gene have been identified in people with permanent neonatal diabetes mellitus (Greeley et al. 2011; Karges et al. 2011; NLM 2014), and mutations are also associated with gestational diabetes mellitus (Zhang et al. 2013). Mutations prevent K(ATP) channels from closing, which leads to reduced insulin secretion from β cells and impaired blood-sugar control. Several environmental chemicals also ranked highly for insulin sensitivity, islet cell, or β cell function based on the KCNJ11 binding assay, including 2,4,6-trichlorophenol, 4-nitrotoluene, ethofumesate and fluometuron. Chemicals with activity profiles to similar to that of tolazamide are shown in Table 4, and the full correlation analysis set is available in Excel File Table S15.

Sulfonylurea herbicides have been used since the early 1980s for the control of nuisance broadleaf weeds and grasses; these herbicides are taken up by the roots and foliage and act by disrupting protein synthesis. They have high toxicity toward plant growth, low application rates, and they are considered to have low toxicity in mammalian studies (Fletcher et al. 1994). General population exposure to sulfonylurea herbicides is not expected to be high because of the low application rates for these herbicides. This assumption is supported by NHANES data showing median urinary levels below the limit of detection for the 17 sulfonylureas included in the biomonitoring program (CDC 2013). Five sulfonylurea herbicides are included in ToxCast™ (under the chemical class “metsulfuron-like”) with generally low rankings except for being in the top ~10% for adipocyte differentiation (thifensulfuron-methyl) and feeding behavior in rodents (flucarbazone-sodium).

**Amitraz.** Amitraz is a formamidine insecticide that has been reported to cause hyperglycemia in children and adults following accidental or deliberate poisoning (“Pesticides Literature Review Documents,” Thayer et al. 2012). The mechanism of action for amitraz as an insecticide is not completely clear but appears to involve alpha-adrenergic agonism, interference with octopamine (the invertebrate equivalent of norepinephrine) action in the central nervous system, uncoupling of oxidative phosphorylation, and inhibition of monoamine oxidases and prostaglandin synthesis [Bonsall and Turnbull 1983; California Environmental Protection Agency (CalEPA) 1995]. Amitraz has also been shown to cause hyperglycemia in dogs (Hsu and Schaffer 1988; Hugnet et al. 1996) and worker honeybees (Cascino et al. 1989) and impaired glucose tolerance in rats (Smith et al. 1990). The hyperglycemia in dogs and the impaired glucose tolerance in rats are accompanied by hypoinsulinemia (Hsu and Schaffer 1988; Hugnet et al. 1996; Smith et al. 1990). The effects of amitraz on glucose are attributed to the activation of α-2 adrenoreceptors, which suppress insulin secretion when activated, presumably through cellular responses that ultimately lead to lower Ca^2+^ concentrations in the cytosol of islet cells (Abu-Basha et al. 1999; Chen and Hsu 1994).

The α-2 adrenergic receptor interactions of amitraz were identified in ToxCast™. The AC_50_ values for amitraz for α-2A and α-2b adrenergic receptors were 0.05–1.6 μM (ADRA2A, Adra2a, Adra2b), but this was not the case for other adrenergic receptor subtypes [α-2C (ADRA2C), β-1 (ADRBI), β-2 (ADRB2), β-3 (ADRB3)]. Amitraz was ranked in the top 10% in both models for feeding behavior and in the model for β cell function (Table 3). Chemicals exhibiting patterns of activity similar to those of amitraz are shown in Table 4, and the full correlation analysis set is available in Excel File Table S16.

**Dexamethasone.** Dexamethasone is a synthetic glucocorticoid that is commonly used to treat inflammatory conditions such as allergic disorders, skin conditions, ulcerative colitis, arthritis, lupus, psoriasis, and breathing disorders. Glucocorticoids cause hyperglycemia, and long-term glucocorticoid therapy has been associated with significant weight gain (Dang et al. 2005; Sheehan 2005). Glucocorticoid receptors (GRs) play a role in committing preadipocytes to the adipocyte lineage and in stimulating adipogenesis (Farmer 2006; Janesick and Blumberg 2011). Dexamethasone was ranked in the top 5% of chemicals in the adipocyte differentiation model but was not ranked highly in any other model (Table 3). Dexamethasone was identified as one of the most potent GR agonists in ToxCast™, and its GR activity was the only factor contributing to its ranking in the adipocyte differentiation model. Chemicals exhibiting patterns of activity similar to that of dexamethasone are shown in Table 4, and the full correlation analysis set is available in Excel File Table S17.

***Signpost chemicals not prioritized in prediction models.*** In some cases, signpost chemicals derived from the peer-reviewed literature were not ranked highly in our analysis, perhaps because the assay targets underlying the response were not selected by our experts, because assays relevant to the mechanism by which the chemical caused the effects were not included in ToxCast™, and/or because potential false negative results in the screening level data were provided by the high-throughput techniques. Understanding the basis for not identifying signpost chemicals is a highly important issue from a public health perspective, where missing active chemicals in a screening strategy is often considered of greater concern than identifying false positives.

**Nicotinic acid (niacin).** Nicotinic acid, or niacin, is a water-soluble B vitamin. At therapeutic doses, it has been associated with hyperglycemia, and at high doses, it can produce hypolipidemia (Dang et al. 2005). This effect appears to be related to increased insulin resistance and to an increase in hepatic gluconeogenesis. None of the models identified niacin as a chemical of concern for metabolic effects (Table 3), perhaps because the relevant assay targets are not included in ToxCast™. The therapeutic effects of niacin are primarily mediated through G protein–coupled receptors not screened in ToxCast™, niacin receptor 1 (NIACR1) and niacin receptor 2 (NIACR2). The niacin receptors have roles in energy regulation (Gille et al. 2008; Hernandez et al. 2010; Mandrika et al. 2010). NIACR1 inhibits cyclic adenosine monophosphate (cAMP) production, which limits fat breakdown in adipose tissue, reducing the amount of free fatty acids available for the liver to produce triglycerides and very-low-density lipoproteins (VLDL) and, consequently, low-density lipoprotein (LDL) or “bad” cholesterol.

**Chlorinated persistent organic pollutants.** A number of chlorinated persistent organic pollutants (POPs) associated with diabetes in humans were tested in ToxCast™ but did not rank highly in our models, including several dichlorodiphenyltrichloroethane (DDT) or dichlorodiphenyldichloroethylene (DDE) isomers (*p*,*p´*-DDE, *p*,*p´*-DDT, *o*,*p´*-DDT), heptachlor epoxide, mirex, dieldrin, β-hexachlorocyclohexane (β-HCH), and lindane (γ-HCH) (Taylor et al. 2013). Of these chemicals, the highest ranked were *o*,*p´*-DDT and β-HCH, which was ranked in the top 15% for rodent feeding behavior based solely on ESRI activity. Similarly, other chlorinated POPs that have not been as well studied for diabetes outcomes in humans were included in ToxCast™ and generally did not rank highly (kepone, endosulfan, endosulfan sulfate, endosulfan I, chlordane, endrin, aldrin, heptachlor, chlorendic acid, *o*,*p´*-DDD, *p*,*p´*-DDD). These chemicals can be identified under the Chemical_Super_Category field in Excel File Tables S4–S8 as “phenol chloro,” “polychloro-bicycle,” and “alkane cyclo chloro.” Our models were not designed to assess many aspects of carbohydrate and lipid metabolism, and additional analysis focusing on these chemicals is an area worthy of future consideration.

***Signpost chemicals not included in the ToxCast™ chemical library.*** A number of chemicals (and their metabolites) that have been most strongly associated with type 2 diabetes in humans have not been tested in the ToxCast™ platforms, including inorganic arsenic species and a number of chlorinated POPs (hexachlorobenzene, oxychlordane, *trans*-nonachlor, PCBs, and dioxins/dioxin-like chemicals) (Kuo et al. 2013; Maull et al. 2012; Taylor et al. 2013).

Another signpost chemical of interest not tested in ToxCast™ is pyrinuron (trade name Vacor), a banned rodenticide associated with type 1 diabetes in humans following acute poisoning episodes (Gallanosa et al. 1981; Karam et al. 1980; Miller et al. 1978; Mindel 1986; Peters et al. 1981; Pont et al. 1979; Prosser and Karam 1978; Yoon 1990). Animal and *in vitro* studies showed that Vacor damaged pancreatic β cells, which led to impaired glucose tolerance in rats (Lee et al. 1988) and to decreased insulin release in isolated rat pancreatic islet cells and hamster insulinoma HIT-T15 cells (Esposti et al. 1996; Taniguchi et al. 1989; Wilson and Gaines 1983). Vacor is a substituted urea compound containing ~ 2% *N*-(3-pyridylmethyl)-*N*´-(*p*-nitrophenyl) urea (PNU, CASRN 53558-25-1) that has been described as causing pancreatic effects similar to those caused by alloxan and streptozotocin (Esposti et al. 1996), two experimental diabetogenic agents that also contain a urea group and were also not included in ToxCast™.

## Discussion

Overall, our analysis suggests that ToxCast™ data can serve as a useful resource for prioritizing chemicals with respect to their potential to alter metabolic function. With the exception of several organotins, the most highly ranked environmental chemicals in the biological process models are not, to our knowledge, being studied for potential metabolic effects. Instead, the research community is focusing on a relatively narrow set of chemicals (or chemical classes) such as bisphenol A, phthalates, perfluorinated chemicals, and certain types of pesticides (Filer et al. 2014).

These results do not demonstrate that the chemicals ranked highest in the models, or considered most similar to signpost chemicals based on correlation analysis, will cause adverse metabolic effects at the organismal level. However, the shortened list of candidates for further testing may increase the feasibility of more time-consuming and expensive follow-up testing to confirm novel metabolic toxicants.

The next steps in considering results from this analysis should include confirming the results presented here with follow-up testing. Additional testing could focus on specific activities (e.g., PPARγ activation) utilizing different technology platforms, or on phenotypic responses using *in vitro* or alternative model systems that align with the biological processes modeled in our analyses (e.g., lipid accumulation in adipocytes, body fat in *C. elegans*, islet cell culture). Follow-up testing is especially important for glycemic control and adipogenic end points because they are understudied in toxicology, making it difficult to systematically evaluate the models presented here with existing data.

Several factors will need to be considered when evaluating the results from follow-up testing. First, binding assays comprise approximately half of the assays used to build the biological process models, in particular for the dopamine, serotonin, and GABA receptor assays used in feeding behavior for *C. elegans* and the β cell function model (see Excel File Table S2). These assays will not provide information about the directionality of activity (i.e., agonist or antagonist), limiting their utility for developing hypotheses about whether a chemical activates or inhibits a biological pathway. For example, several antidiabetogenic drugs were identified as active for islet or β cell function and insulin sensitivity in peripheral tissue. Similarly, RAR agonist activity needs to be considered when evaluating the results from the adipocyte differentiation model. Activation of RAR is associated with an antiadiposity phenotype (Bonet et al. 2012; Frey and Vogel 2011), although it should be noted that impaired adipogenesis can itself be metabolically deleterious because failure to expand adipose depots (e.g., in clinical states of lipodystrophy) promotes insulin resistance and diabetes.

Second, there is concern about the specificity of gene-based assays in the context of cytotoxicity and other secondary mechanisms leading to potential false positive results. To limit the influence of cytotoxicity and other secondary mechanisms in the models, we weighted the input data based on the distribution of cytotoxicity assays, down-weighting and often removing data for lack of specificity. Using this approach biases the analysis toward identifying chemicals that are specific for the assay targets of interest. Consequently, chemicals that exhibit broad-spectrum toxicity at low concentrations, such as tributyltin chloride, will not rank as highly as if less-stringent cytotoxicity filtering were used.

Third, a number of gene targets identified by experts during the 2011 NTP workshop as relevant to the biological processes described in this article are not included in ToxCast™, including glucose transporter 2 (GLUT2), insulin receptor substrates 1 and 2 (IRS1, IRS2), the ZFP423 gene and Wnt genes involved in adipogenesis, leptin receptor (LEPR), fatty acid binding protein 4 (FABP4, found in adipocytes), and genes expressed in stem cells that populate white adipose tissue lineage and could be early indicators of commitment to adipocyte lineage (CD24, CD29, CD34, PDGFRb, NG2, Sca1).

Finally, there is limited metabolizing capability in both the Tox21 and ToxCast™ platforms. The chemical library contains key metabolites for limited chemicals: for example, metabolites of phthalates and organophosphate pesticides. However, it is likely that many other *in vitro* screens will have the same limitation.

Despite the limitations in using ToxCast™ HTS data, it is encouraging that the models identified the majority of the signpost chemicals for metabolic effects including amitraz, tributyltins, nicotine, and several drugs. In this analysis, we relied exclusively on expert opinion to identify relevant assays for our models. We considered selecting assay targets based on bioinformatics-based biological process/pathway databases, such as Kyoto Encyclopedia of Genes and Genomes (KEGG) (Kanehisa et al. 2014) or CoPub (Frijters et al. 2008), but we decided to utilize expert opinion for several reasons. First, the gene coverage of biological pathways within ToxCast™ varies and is limited for pathways related to diabetes and obesity. For example, the KEGG pathway for “Type II diabetes mellitus–Homo sapiens (human)” includes > 50 genes, but approximately half of these are not included in ToxCast™ assays. Second, genes identified in text-mining resources such as CoPub do not indicate the directionality of the association with the biological process (i.e., activation/antagonism or up-/down-regulation), which is important when trying to identify assay targets associated with potentially adverse health outcomes. Third, the gene targets identified from the pathway databases might not necessarily be applicable to specific medium-throughput methods that could be used to assess the results, including *in vitro* models of islet/β cell function, adipocyte differentiation, and feeding behavior and body fat in *C. elegans*. In other words, the relevance of different assays may differ depending on the model system used in more targeted research. We do not consider this a shortcoming of our analysis; instead, it reflects a practical approach to using HTS. Future analyses of this type could perhaps be improved by using a combination of approaches including the use of expert opinion, performing systematic reviews of the literature to identify signpost chemicals for mechanistic insight, and utilization of bioinformatics-based databases such as KEGG and CoPub. ToxCast™ data can also be used to complement other databases developed to annotate gene interactions of environmental chemicals such as the Comparative Toxicogenomics Database (CTD, Davis et al. 2013) and the Pesticide Target Interaction Database (PTID, Gong et al. 2013).

Analogous to the biological process scores, we used the *z*-score values to calculate the chemical–chemical correlations. Anchoring the correlation analysis to *z*-score values identifies chemicals with similar specific profiles despite shifts in potency, allowing us to identify environmental chemicals similar to the signpost chemicals even though they may often have lower potency values. In this sense, use of the *z*-scores in similarity profiling can identify potential health outcomes. The dose level at which an effect occurs (i.e., the potency), particularly within the context of potential exposure, also needs to be considered. Using AC_50_ values (concentration at half-maximal activity) in the correlation analysis would better capture the potential potency of an environmental chemical but would likely remove the specificity of any similarity. For completeness, we present the results of the correlation analysis based on AC_50_ data in Excel File Tables S9–S17 because using the AC_50_ data did alter the ranking of chemicals considered most similar.

The clustering presented in Figure S7 illustrates one approach to assessing profile similarity, although ToxPi output data are provided in the supplemental tables to facilitate alternative approaches. Nevertheless, the *C. elegans* feeding behavior clusters illustrate the notion of chemical “activity” as a multidimensional phenomenon. Across diverse compound and assay sets, different components of activity will come to the fore, which is why ToxPi scores should always be interpreted in context with slice-wise profiles.

## Conclusions

The results of this screening-level analysis suggest that the spectrum of environmental chemicals to consider in research related to diabetes and obesity is much broader than indicated from research papers and reviews published in the peer-reviewed literature. Certainly, additional research is required to put these screening-level analyses into context, but our hope is that the information presented in this review facilitates the development of new hypotheses by researchers interested in understanding the potential role of environmental chemicals in the development or progression of disease for diabetes, obesity, and metabolic syndrome.

## Supplemental Material

(4.5 MB) PDF

(2.5 MB) ZIP
