# Supplementary material for: Prioritizing Environmental Chemicals for Obesity and Diabetes Outcomes Research: A Screening Approach Using ToxCast™ High-Throughput Data
Source: Environ Health Perspect. 2016 Mar 15;124(8):1141–54. doi: 10.1289/ehp.1510456 (PMC4977057; doi:10.1289/ehp.1510456)

Environ Health Perspect
DOI: 10.1289/ehp.1510456

Note to readers with disabilities: *EHP* strives to ensure that all journal content is accessible to all readers. However, some figures and Supplemental Material published in *EHP* articles may not conform to 508 standards due to the complexity of the information being presented. If you need assistance accessing journal content, please contact ehp508@niehs.nih.gov. Our staff will work with you to assess and meet your accessibility needs within 3 working days.

Supplemental Material

Prioritizing Environmental Chemicals for Obesity and Diabetes Outcomes Research: A Screening Approach Using ToxCast High Throughput Data

Scott Auerbach, Dayne Filer, David Reif, Vickie Walker, Alison C. Holloway, Jennifer Schlezinger, Supriya Srinivasan, Daniel Svoboda, Richard Judson, John R. Bucher, and Kristina A. Thayer

Table of Contents

Figures S1-S6. Top 30 chemicals in prediction models for adipocyte differentiation, feeding behavior (rodent), and feeding behavior (*C. elegans*), insulin sensitivity, and islet cell function. Screening for identifying chemicals with potential metabolic effects based on overall biological process score. (a) The ToxPi for the highest scoring 30 chemicals in the iCSS Dashboard, plotted in decreasing order by their respective biological process score. Each ToxPi represents one chemical, with the chemical name near the ToxPi and biological process score in parentheses. The CAS number and information on usage and chemical class is also presented when known. (b) The distribution of scores for all 1860 chemicals in the model. Blue points highlight the location of environmental chemicals already being studied for metabolic effects listed on the y-axis, and the red points highlight the chemicals listed in A relative to the whole chemical library.

Figure S1. Adipocyte differentiation

Figure S2. Feeding behavior (rodents)

Figure S3. Feeding behavior (*C. elegans*)

Figure S4. Insulin sensitivity

Figure S5. Islet cell function

Figure S6. Beta cell function

Figures S7-S12. Dendrograms representing hierarchy of structural similarity among ToxPi Top 30 lists of chemicals. The Tox21 set of chemicals (8307 InChIS) were imported into a Leadscope Enterprise v3.2 project, and subsequently exported as Leadscope "fingerprints". A chemical's fingerprint consists of a bitmap representing the presence or absence of ~27000 structural features. Fingerprints were extracted from the entire Tox21 set for each "top 30" list (Excel File Table S18). Features not present in any of the 30 members comprising a given list were eliminated, yielding fingerprints characterized by 611 – 843 chemical features. Fingerprints from ToxPi "top 30" lists were clustered (single linkage; tanimoto), yielding dendrograms, similarity scores, and heatmaps. Structure-activity classes are elucidated by inspecting line graphs of the tanimoto coefficients, plotted coincident with the dendrograms. Heatmaps (blue = feature present; grey = absent) align structural features common among the chemical sets. There are 30 chemicals in each structural hierarchy, having 29 similarity coefficients, each representing the structural similarity between nearest neighbors. Each dendrogram and similarity profile, together with an understanding of the biological effects of certain chemicals, enabled elucidation of number of enriched chemical classes within each of several "top 30" lists. Heatmaps contain alignments of structural features common among chemicals in the lists (not described here). Figures S7-S12 reveal chemical classes present in 3 of the 6 ToxPi Top 30 lists. Rodent feeding behavior (Figure S8) is clearly affected by both estrogens, and non-steroidal estrogenic compounds. Adipocyte differentiation (Figure S7) collects tin containing chemicals, along with glucocorticoids, and CPs. Finally, Islet Cell Model 2 (Figure S12) is clearly affected by estrogens (see also, Excel File Table S18). The remaining ToxPi top 30 lists reveal no discernible chemical classes.

Figure S7. Adipocyte differentiation

Figure S8. Feeding behavior (rodent)

Figure S9. Feeding behavior (*C. elegans*)

Figure S10. Insulin sensitivity

Figure S11. Islet cell function

Figure S12. Beta cell function

Figure S13. Chemical clustering based upon model of feeding behavior in *C. elegans*. The first three principal components (PCs) from the ToxPi model of feeding behavior in *C. elegans* are plotted in a pair-wise matrix of each PC. The points, each representing a single chemical, are colored according to the k-means clustering of the PC output. The three cluster insets show the mean ToxPi profile (plus overall ToxPi score) for chemicals in that cluster. The component assays in each slice are indicated in Table 1.

Additional Files

Supplemental Code and Data Zip File

Supplemental Code and Data Zip File Index

Excel File Tables S1-S18

Excel File Table S1. Description of Phase 2 ToxCast assays

Excel File Table S2. Phase 2 ToxCast assays used in prediction models

Excel File Table S3. Prediction model for adipocyte differentiation

Excel File Table S4. Prediction model for feeding behavior in rodents

Excel File Table S5. Prediction model for feeding behavior in *C. elegans*

Excel File Table S6. Prediction model for insulin sensitivity

Excel File Table S7. Prediction model for islet cell function

Excel File Table S8. Prediction model for pancreatic beta cell function

Excel File Table S9. Correlation analysis for chemicals having similar activity to troglitazone

Excel File Table S10. Correlation analysis for chemicals having similar activity to tributyltin chloride

Excel File Table S11. Correlation analysis for chemicals having similar activity to tributyltin methacrylate

Excel File Table S12. Correlation analysis for chemicals having similar activity to nicotine

Excel File Table S13. Correlation analysis for chemicals having similar activity to haloperidol

Excel File Table S14. Correlation analysis for chemicals having similar activity to chlorpromazine

Excel File Table S15. Correlation analysis for chemicals having similar activity to tolazamide

Excel File Table S16. Correlation analysis for chemicals having similar activity to amitraz

Excel File Table S17. Correlation analysis for chemicals having similar activity to dexamethasone

Excel File Table S18. Chemical structures represented in the top 30 lists

R Scripts

- correlation_analysis.R
- generate.allmodels.input.r
- generate.inputs_v5.r
- generate.inputs_v6.r
- generate.inputs_v7.r
- generate.inputs_v8.r
- mapcheminfo.r
- ToxPi
  - Scripts
    - color.select.ind.R
    - color.select.R
    - data.parse.R
    - easy.colors.R
    - implement_vNIEHS.R
    - refchemlabel.R
    - server.implement.R
    - toxpi4_vNIEHS.R
- ToxPi_Combined_140801.r
- ToxPi_Models_140801.r
- ToxPi_Models_150428.r

Figure S1. Adipocyte Differentiation

(a)

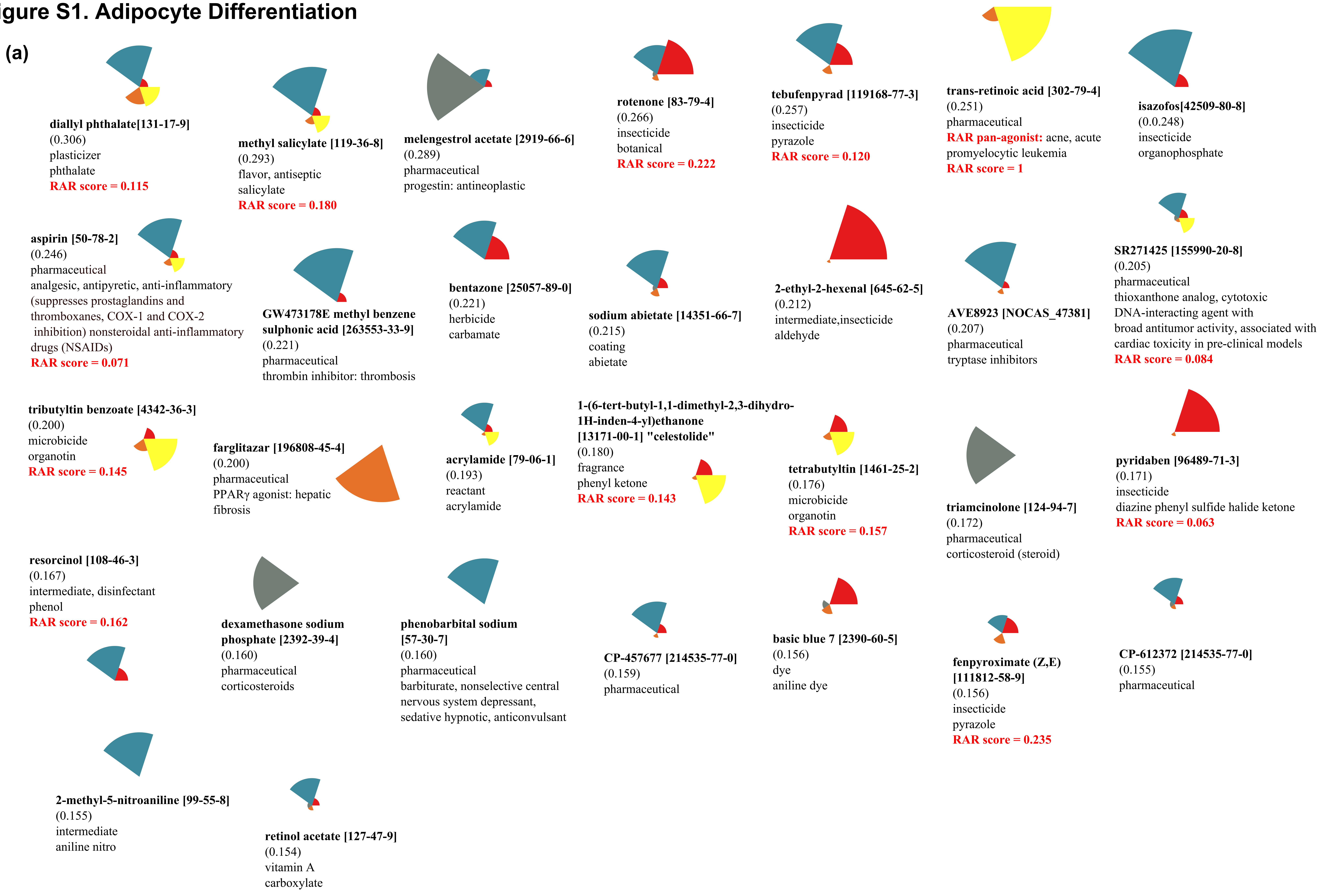

(b)

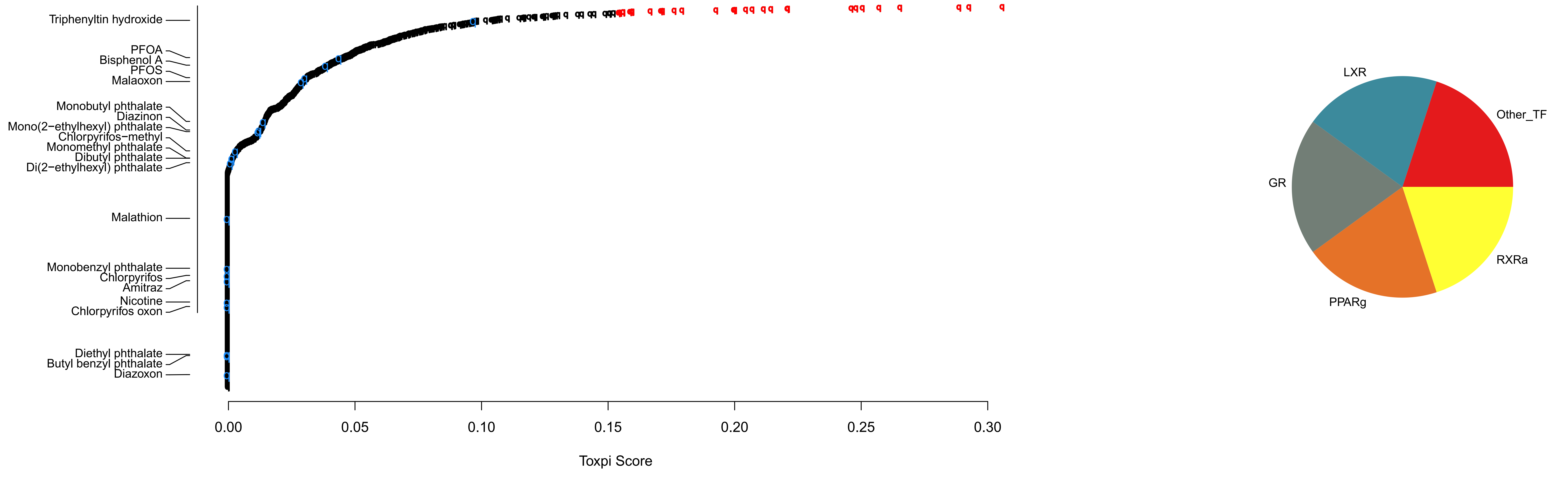

Figure S2. Feeding Behavior (rodents)

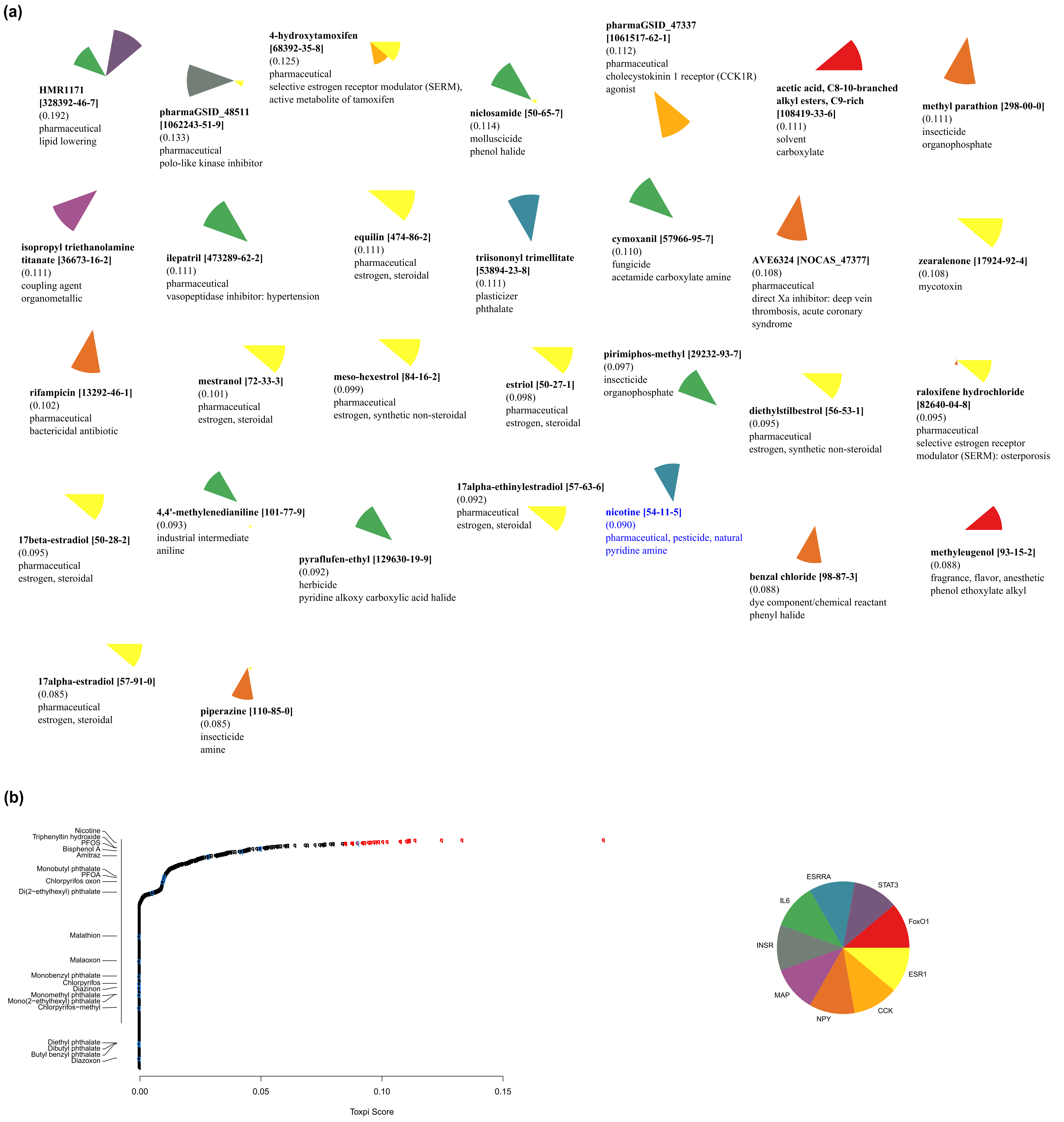

Figure S3. Feeding Behavior (C. elegans)

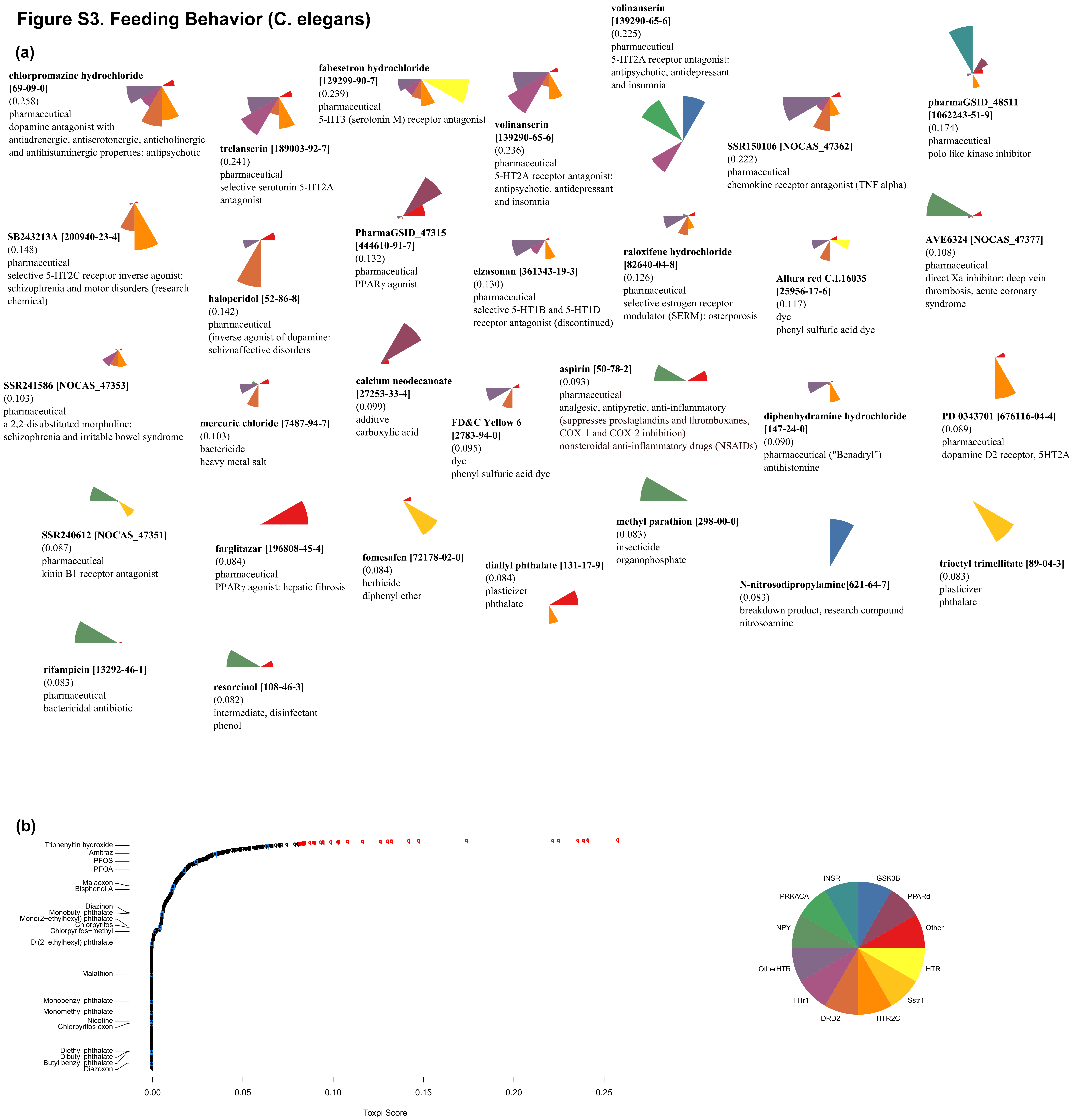

Figure S4. Insulin Sensitivity

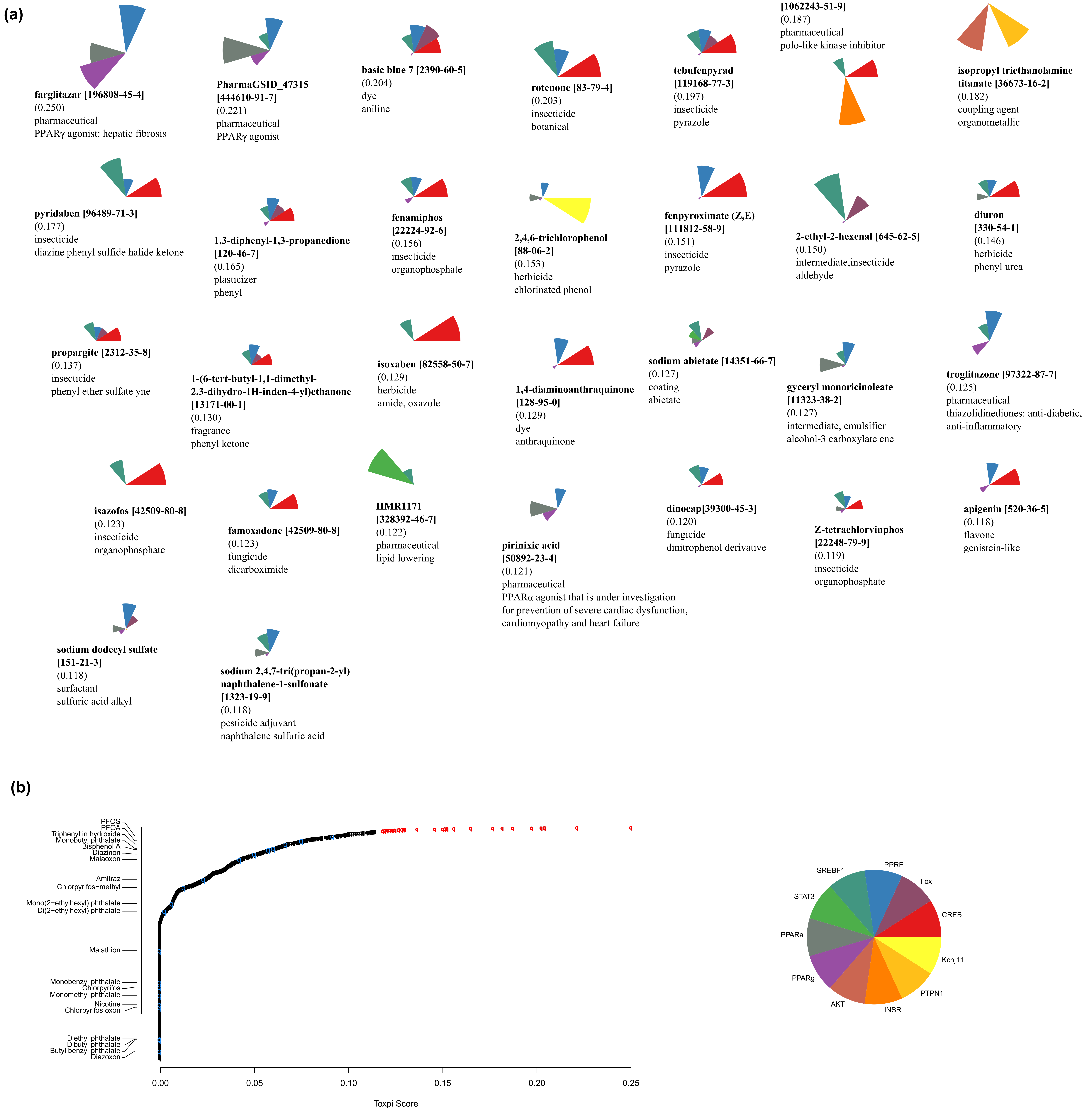

Figure S5. Islet Cell Function

(a)

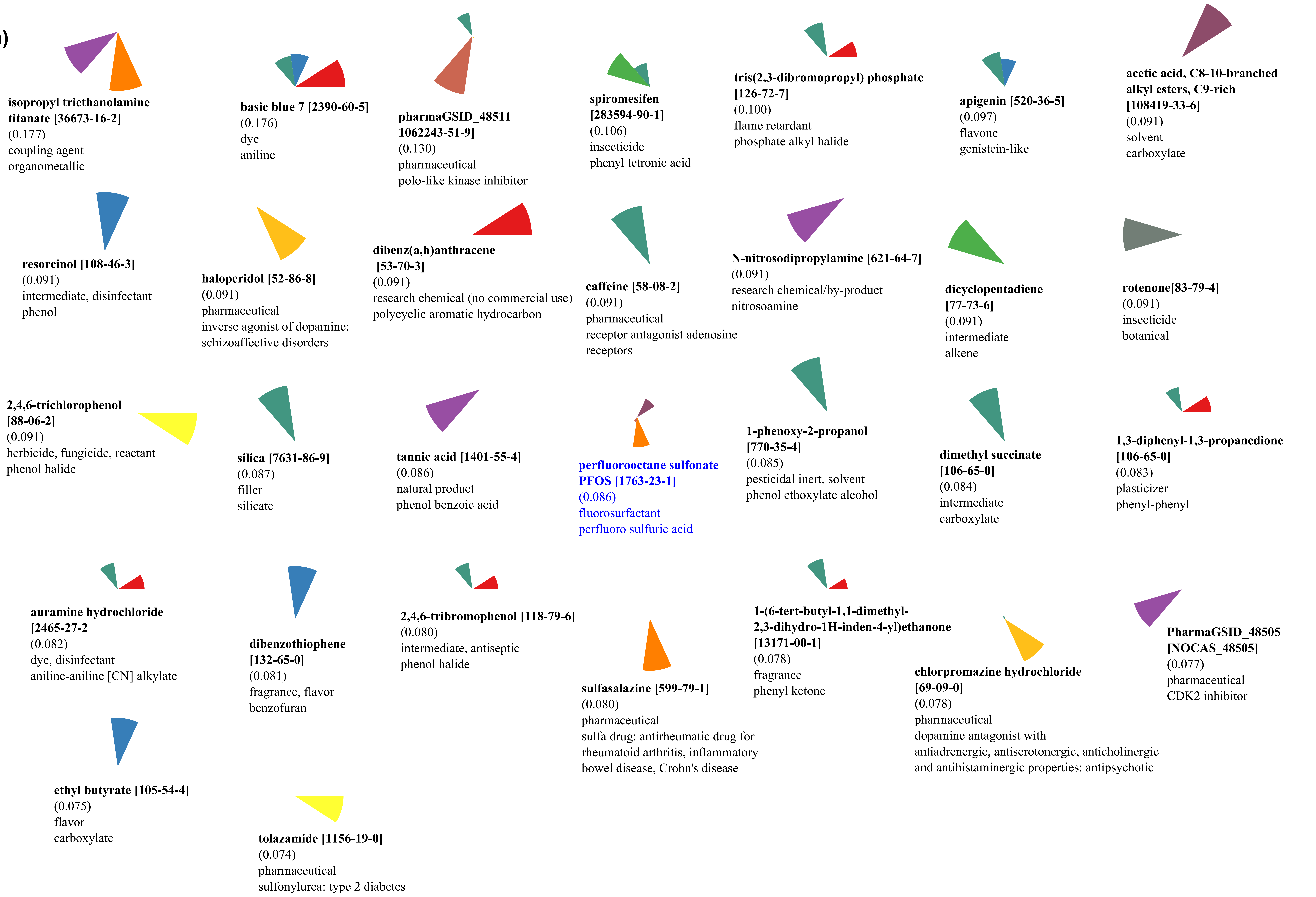

(b)

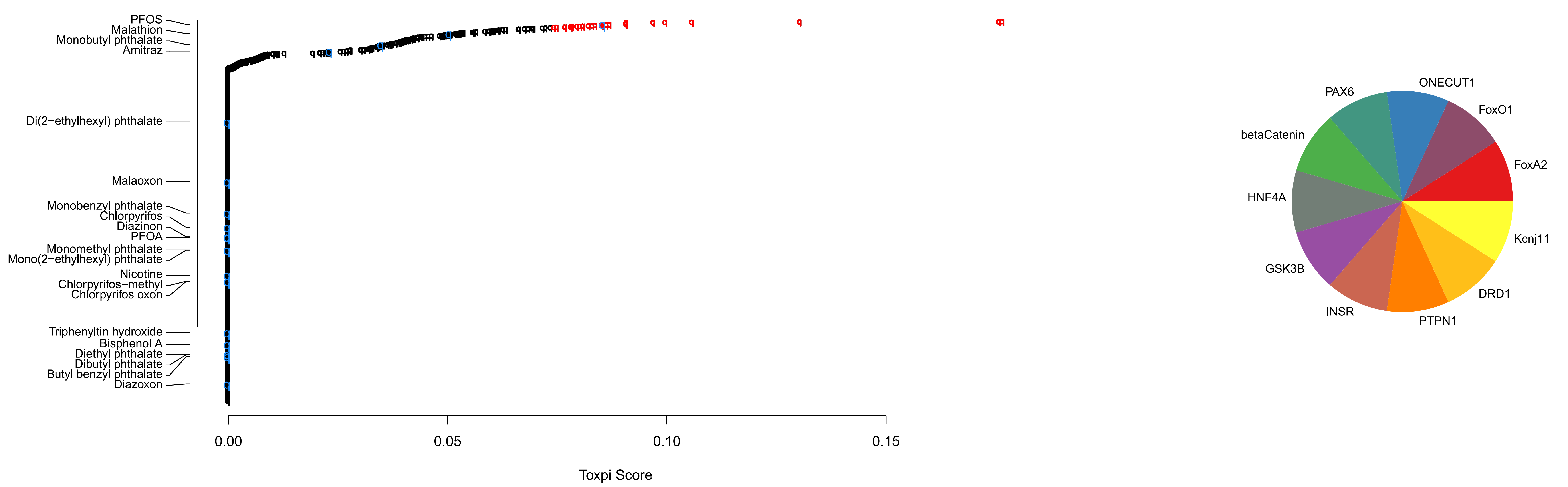

Figure S6. Beta Cell Function

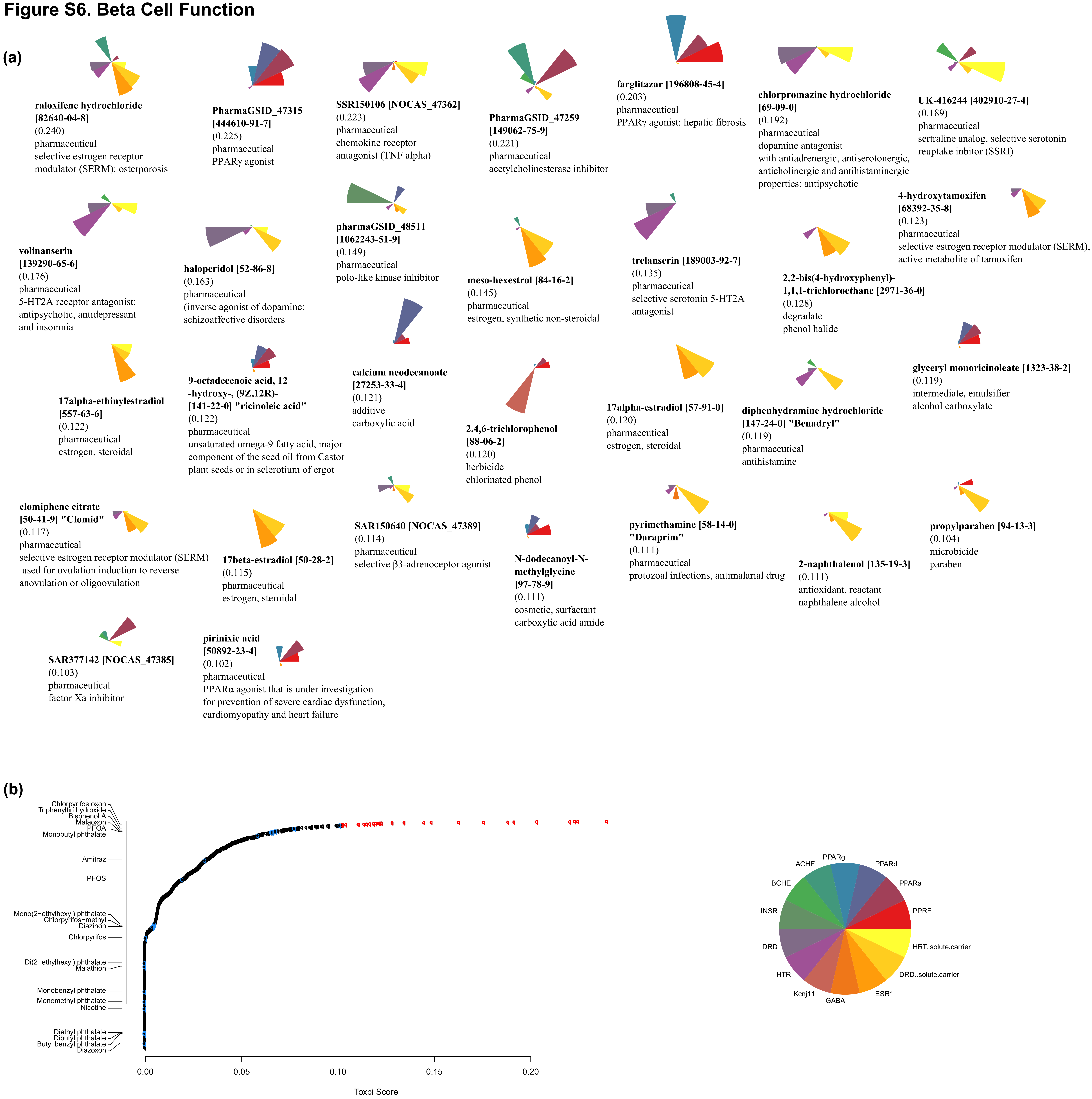

Figures S7-S12. Dendrograms representing hierarchy of structural similarity among ToxPi Top 30 lists of chemicals

The Tox21 set of chemicals (8307 InChIS) were imported into a Leadscope Enterprise v3.2 project, and subsequently exported as Leadscope "fingerprints". A chemical's fingerprint consists of a bitmap representing the presence or absence of ~27000 structural features. Fingerprints were extracted from the entire Tox21 set for each "top 30" list (Excel File Table S18). Features not present in any of the 30 members comprising a given list were eliminated, yielding fingerprints characterized by 611 – 843 chemical features. Fingerprints from ToxPi "top 30" lists were clustered (single linkage; tanimoto), yielding dendrograms, similarity scores, and heatmaps. Structure-activity classes are elucidated by inspecting line graphs of the tanimoto coefficients, plotted coincident with the dendrograms. Heatmaps (blue = feature present; grey = absent) align structural features common among the chemical sets. There are 30 chemicals in each structural hierarchy, having 29 similarity coefficients, each representing the structural similarity between nearest neighbors. Each dendrogram and similarity profile, together with an understanding of the biological effects of certain chemicals, enabled elucidation of number of enriched chemical classes within each of several "top 30" lists. Heatmaps contain alignments of structural features common among chemicals in the lists (not described here). Figures S7-S12 reveal chemical classes present in 3 of the 6 ToxPi Top 30 lists. Rodent feeding behavior (Figure S8) is clearly affected by both estrogens, and non-steroidal estrogenic compounds. Adipocyte differentiation (Figure S7) collects tin containing chemicals, along with glucocorticoids, and CPs. Finally, Islet Cell Model 2 (Figure S12) is clearly affected by estrogens (see also, Excel File Table S18). The remaining ToxPi top 30 lists reveal no discernible chemical classes.

Figure S7. Adipocyte Differentiation

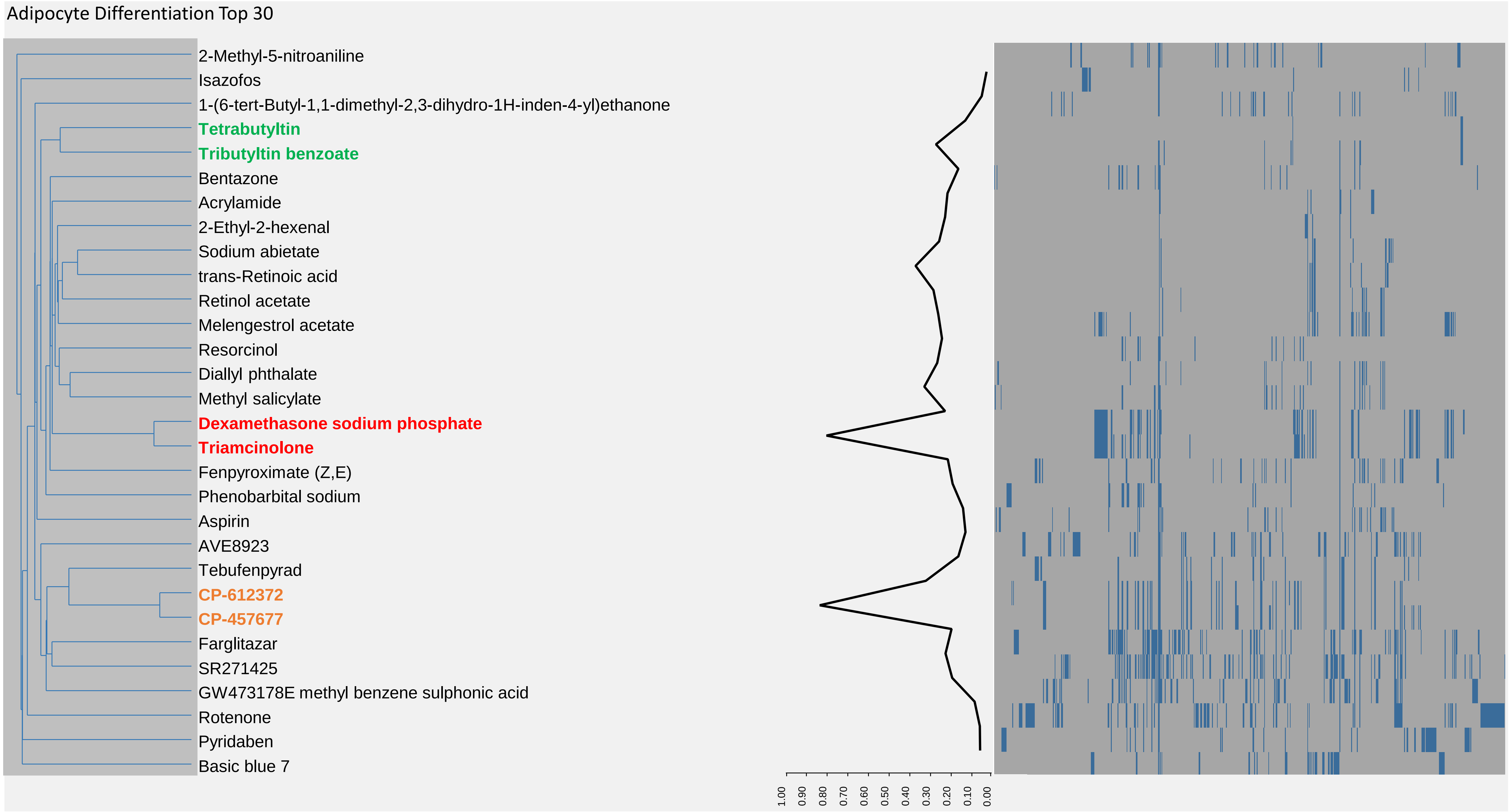

Figure S8. Feeding Behavior (rodent)

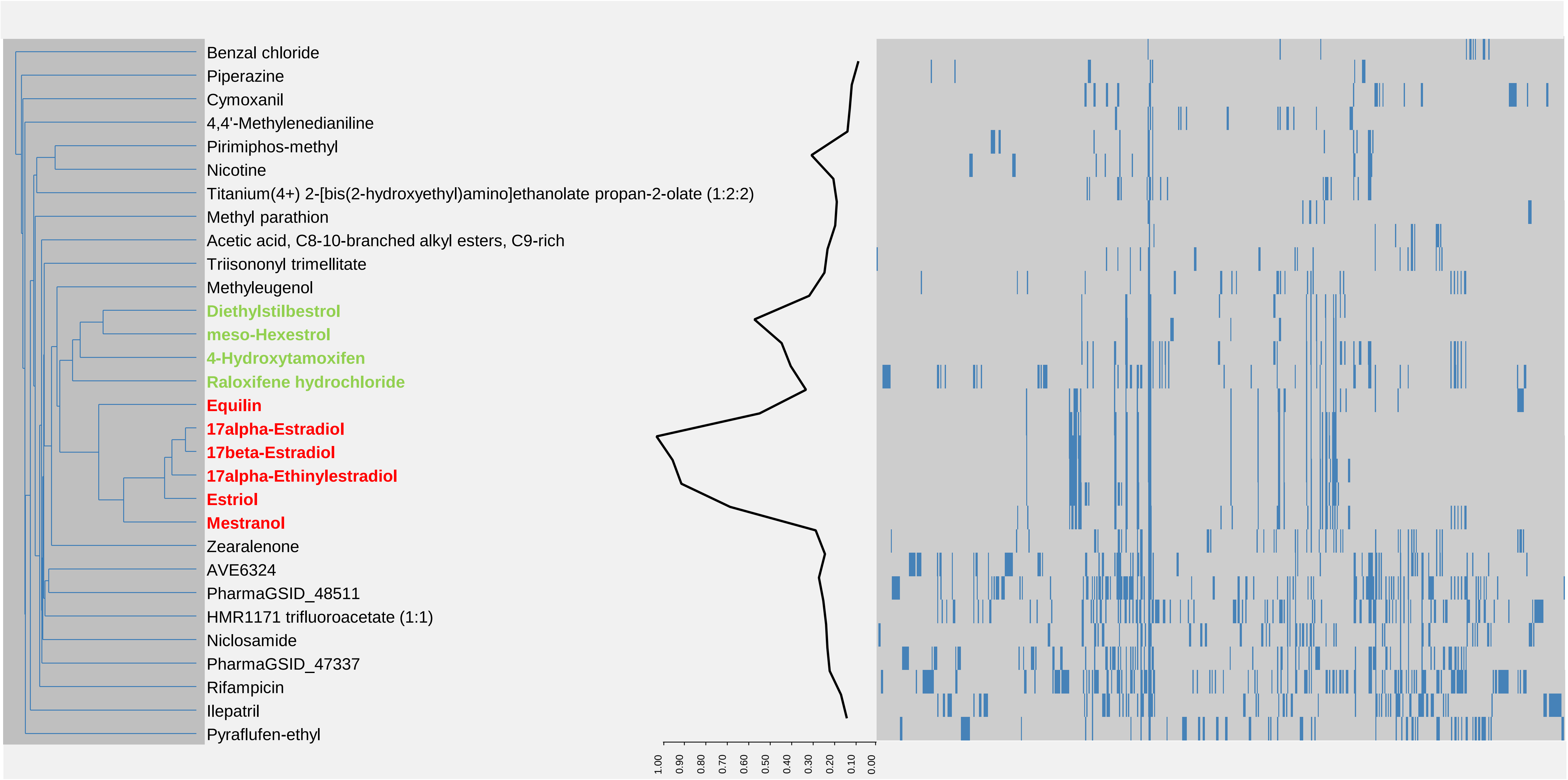

Figure S9. Feeding Behavior (*c. elegans*)

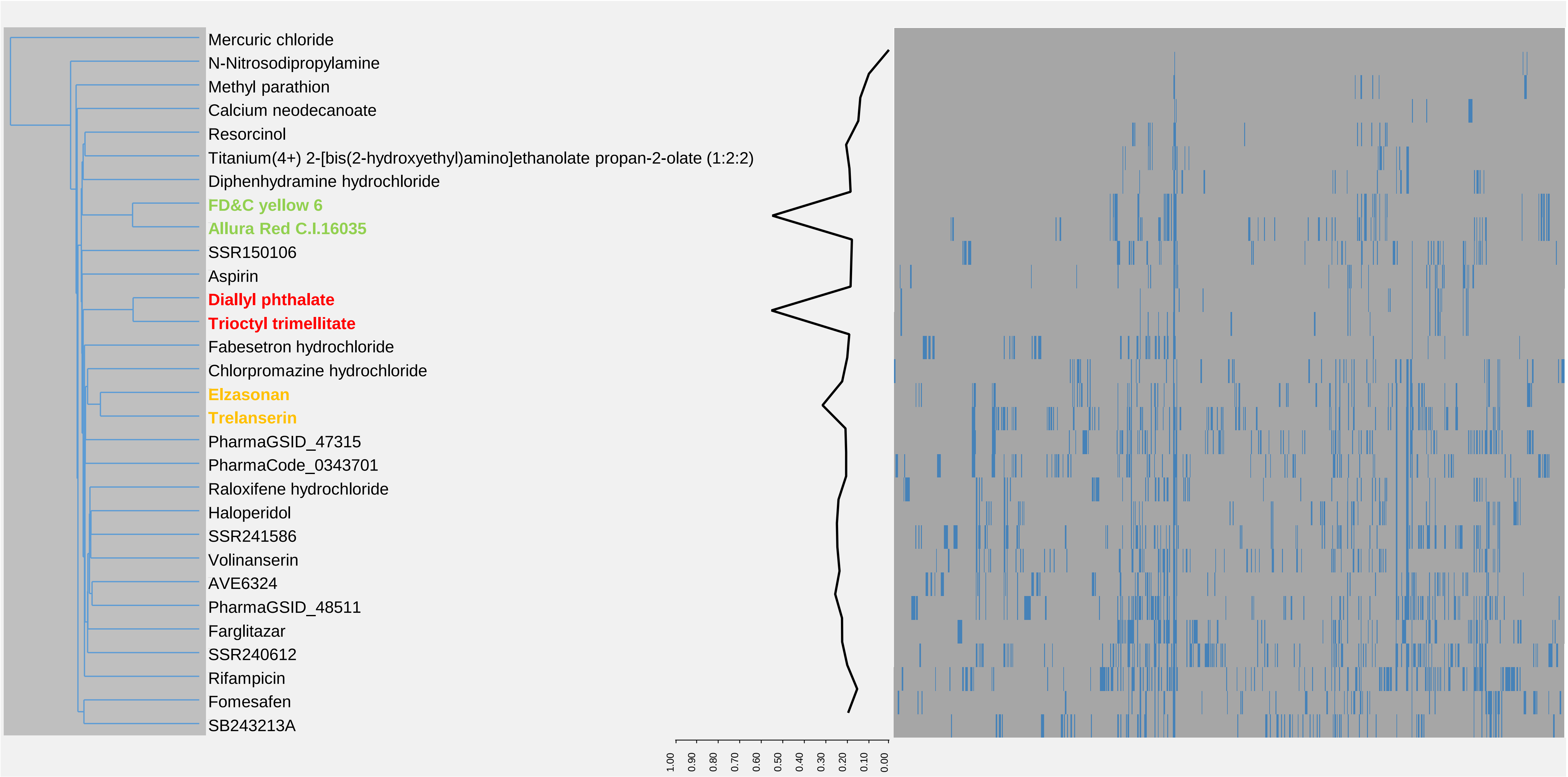

Figure S10. Insulin Sensitivity

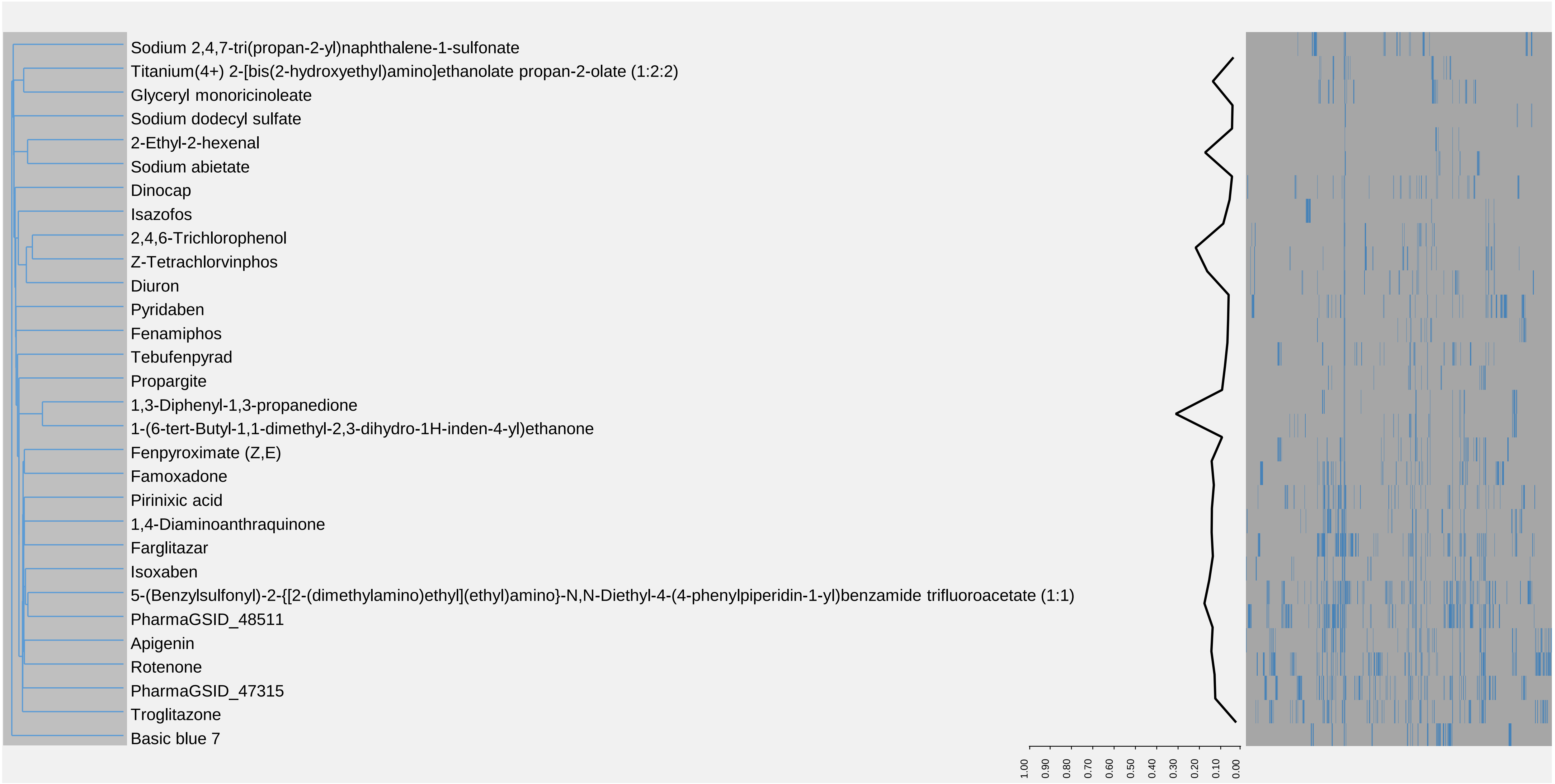

Figure S11. Islet Cell Function

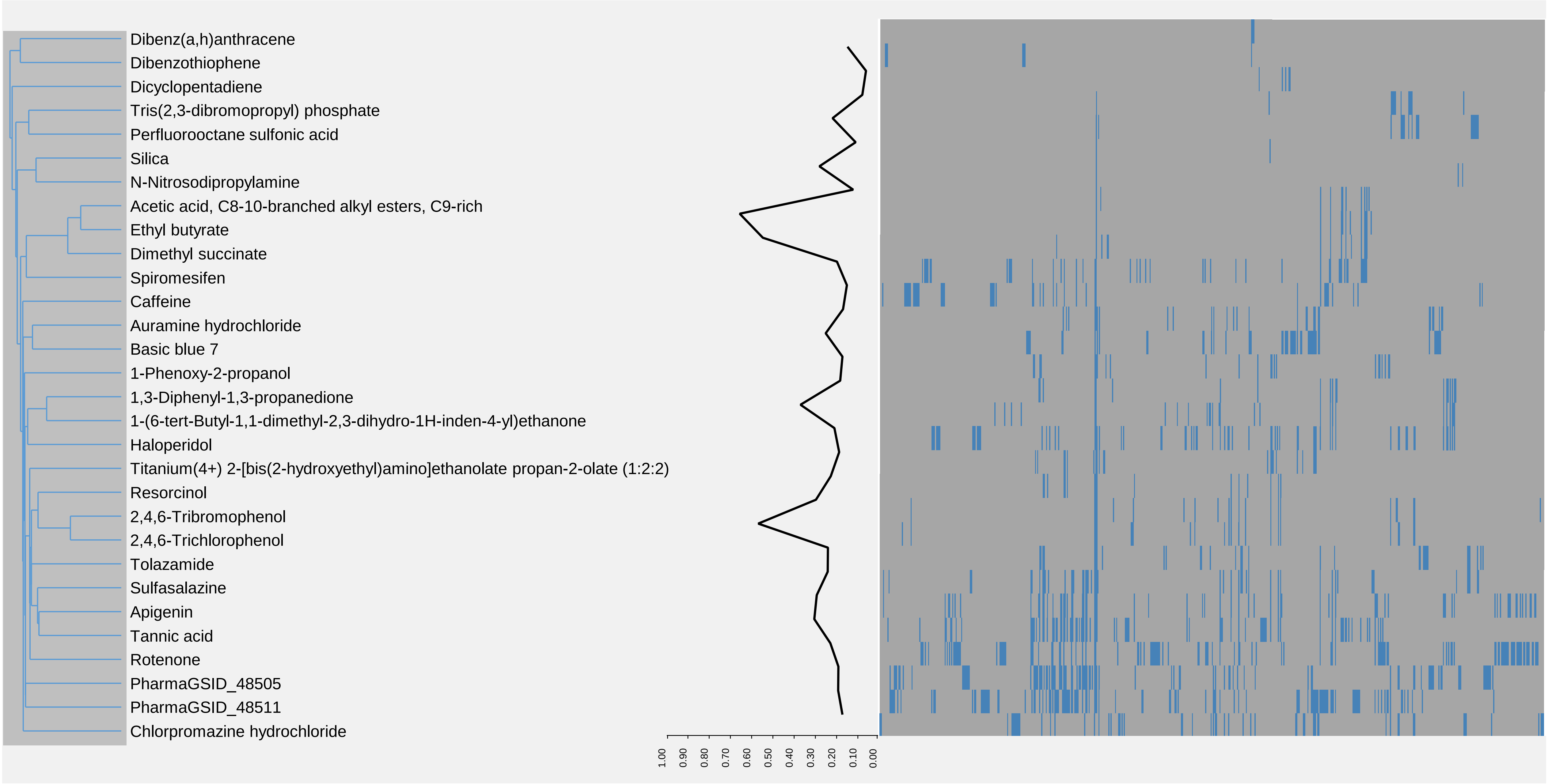

Figure S12. Beta Cell Function

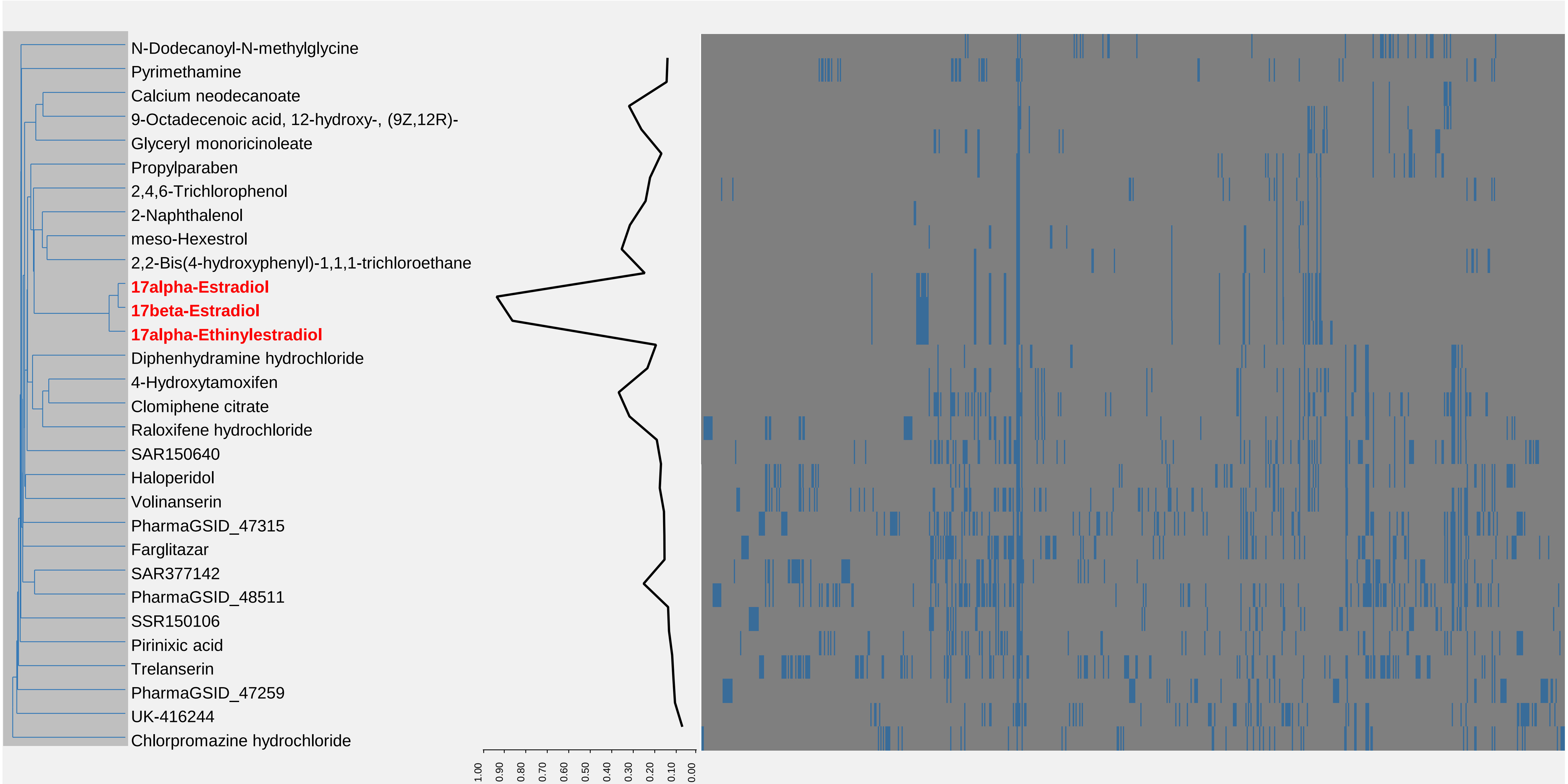

Figure S13. Chemical clustering based upon model of feeding behavior in C. elegans

The first three principal components (PCs) from the ToxPi model of feeding behavior in C. elegans are plotted in a pair-wise matrix of each PC. The points, each representing a single chemical, are colored according to the *k*-means clustering of the PC output. The three cluster insets show the mean ToxPi profile (plus overall ToxPi score) for chemicals in that cluster. The component assays in each slice are indicated in Table 1.

Figure S13. Chemical clustering based upon model of feeding behavior in C. elegans

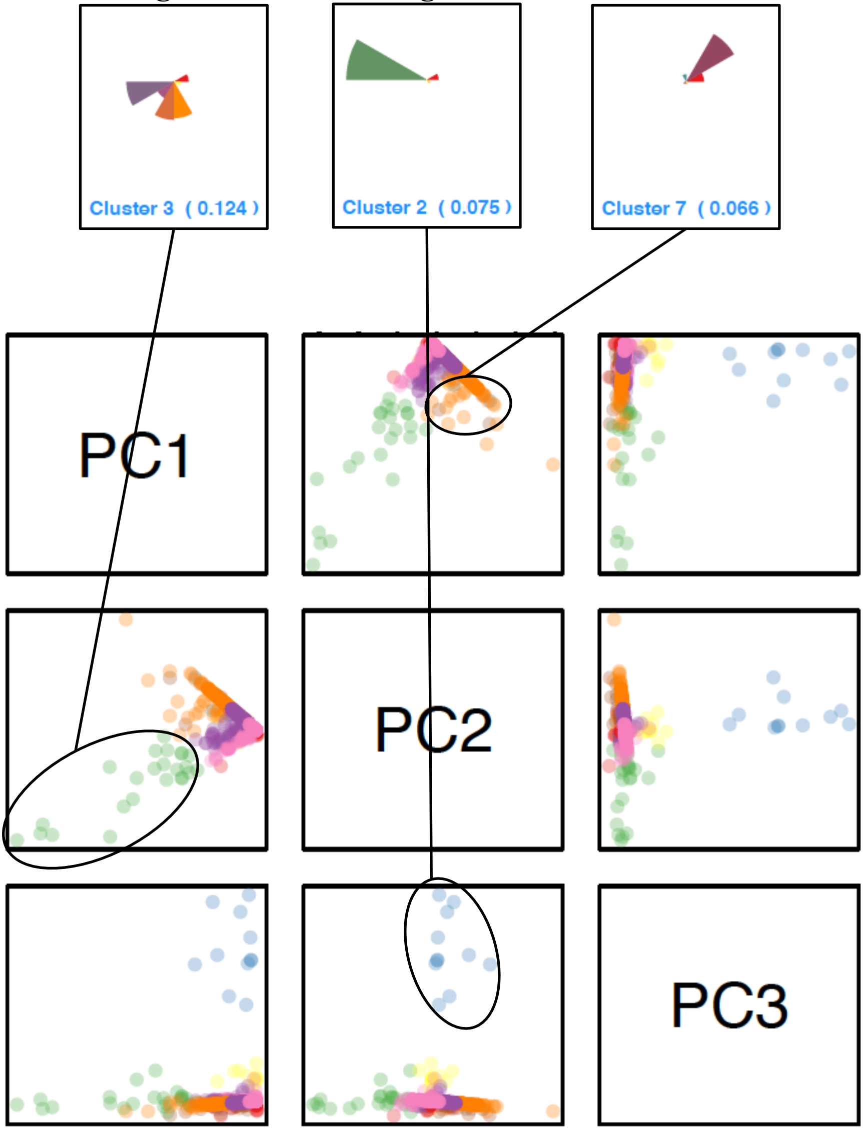

Supplement: (4.5 MB) PDF [file ehp.1510456.s001.acco.pdf]
